# Association between intake of different beverages and risk of hyperuricemia and gout: a risk factor and dose–response meta–analysis

**DOI:** 10.3389/fnut.2026.1956907

**Published:** 2026-10-22

**Authors:** Mengxiao Li, Jiaying Wang, Zixi Wang, Jiaoyan Li, Wei Li

**Affiliations:** Department of Nephrology, Beijing University of Chinese Medicine Third Affiliated Hospital, Beijing, China

**Keywords:** beverages, dose–response, gout, hyperuricemia, meta-analysis

## Abstract

**Background:**

The purpose of this study is to thoroughly review and compile evidence regarding the relationship between the consumption of various drinks and the risk of developing gout and hyperuricemia (HUA).

**Methods:**

We conducted a comprehensive search of PubMed, Embase, the Cochrane Library, and Web of Science to find cross-sectional, cohort, and case–control studies examining the link between the consumption of various beverages and HUA or gout. Fixed-effects models and random-effects models were used for the meta-analysis. Stata 18.0 was employed for meta-analysis and dose–response analysis to determine pooled odds ratios (ORs) and hazard ratios (HRs) with 95% confidence intervals (CIs). Statistical heterogeneity was quantified using the I^2^ statistic. Sensitivity analyses were performed using the leave-one-out method. Publication bias was assessed by funnel plot inspection and Egger’s test.

**Results:**

The study included a total of 52 articles. The meta-analysis showed that alcohol intake (OR = 1.809, 95% CI: 1.372–2.385, *p* < 0.001 for gout; OR = 1.524, 95% CI: 1.363–1.703, *p* < 0.001 for HUA) and intake of sugar-sweetened beverages (SSBs) (OR = 1.371, 95% CI: 1.059–1.776, *p* = 0.017 for gout; OR = 1.478, 95% CI: 1.342–1.627, *p* < 0.001 for HUA) were connected to an elevated risk of gout and HUA, and at conventional intake levels, tea was associated with an increased risk of HUA among female (OR = 1.294, 95% CI: 1.081–1.549, *p* = 0.005) subgroups. Coffee intake was linked to a reduced risk of developing gout (OR = 0.543, 95% CI: 0.341–0.866, *p* = 0.010). Analyses of dose–response revealed a negative correlation between coffee consumption and gout, while alcohol and sugar-sweetened beverages were positively linked to both gout and hyperuricemia. Nonlinear relationships were found between alcohol consumption and gout, as well as between sugar-sweetened beverage consumption and hyperuricemia, but not for the other overall associations. These results were exploratory due to the limited number of studies included in each analysis. Substantial between-study heterogeneity was observed across most analyses, reflecting variation in study design, population characteristics, and exposure definitions. Leave-one-out sensitivity analyses typically indicated consistent association directions, but the statistical significance was affected by which studies were included for certain outcomes. Given the relatively small number of studies included in most analyses, Egger’s test may have had insufficient statistical power to reliably detect funnel plot asymmetry. The publication bias assessments reported here are therefore exploratory in nature, and the possibility of small-study effects or selective reporting cannot be fully excluded.

**Conclusion:**

Alcohol and SSB intake were associated with higher odds of gout and HUA. Current research indicates that there is an association between coffee intake and a reduced risk of gout, while tea consumption may be associated with an increased risk of HUA in female subgroups, though this requires further validation.

**Systematic review registration:**

https://www.crd.york.ac.uk/prospero/, identifier CRD420261279131.

## Introduction

HUA is a metabolic disorder marked by high uric acid levels in the blood, caused by faulty purine metabolism or reduced uric acid excretion (1). Gout is a consequence of the deposition of urate crystals in joints and renal tissues, which induces inflammation and tissue damage, and is associated with abnormalities in purine metabolism (2). The global prevalence and incidence of HUA and gout have markedly increased, driven by rapid economic development worldwide and concurrent changes in dietary and lifestyle habits. Recent comprehensive analyses from the Global Burden of Disease study highlight this rising trend as a significant public health challenge (3). Beyond its role in precipitating gout, HUA independently elevates the risk of chronic kidney disease, cardiovascular disease, type 2 diabetes, and ischemic stroke (4). Consequently, HUA is increasingly acknowledged as a significant metabolic disorder, comparable to hypertension, hyperglycemia, and hyperlipidemia, posing substantial clinical and economic challenges to global public health systems. A meta-analysis indicates that individuals suffering from gout have a considerably increased mortality risk from cardiovascular and coronary heart diseases compared to the general population (5). Therefore, it is imperative to explore modifiable lifestyle factors, particularly focusing on daily beverage consumption and its influence on uric acid metabolism, to reduce the prevalence of HUA, gout, and related conditions.

Different beverages, due to their varied chemical compositions, have distinct biological effects on purine synthesis, the production of uric acid, and the mechanisms of renal excretion (6). For example, alcohol increases uric acid levels by augmenting the hepatic breakdown of adenosine triphosphate (ATP). Concurrently, its metabolite, lactic acid, competes with uric acid for renal tubular excretion pathways, thereby inhibiting uric acid clearance. Notably, beer is rich in highly bioavailable purines, such as guanosine, which further exacerbates the risk of HUA (7). The high fructose content in SSBs is rapidly phosphorylated by hepatic fructokinase, leading to the depletion of intracellular ATP. This process directly promotes uric acid synthesis and may indirectly impair renal clearance by inducing insulin resistance (8). Conversely, numerous cohort studies suggest that coffee consumption correlates with a reduced risk of developing gout. This protective effect could be mediated by caffeine-mediated antagonism of adenosine receptors, improvements in insulin sensitivity induced by chlorogenic acid, and enhanced renal excretion (9). Similarly, tea extracts have been demonstrated to suppress xanthine oxidase activity and decrease oxidative stress in animal studies, indicating a potential protective effect on uric acid metabolism (10).

Despite extensive research into the biological connections between beverage consumption and uric acid metabolism, earlier epidemiological findings remain inconsistent. Moreover, an updated synthesis is needed to assess whether associations between common beverage categories and gout or hyperuricemia remain consistent across outcomes and subgroups. Differences in exposure definitions also warrant explicit consideration when examining dose–response relationships across studies.

This research aims to comprehensively investigate the consumption of various beverages and their associations with the probability of developing gout and HUA, as well as to quantify the dose–response relationships between different beverage intakes, HUA, and gout through a meta-analysis. By synthesizing global cross-sectional, case–control, and cohort data while adhering to rigorous methodological standards, the study examined nonlinear associations, thereby providing robust evidence to inform refined dietary management and prevention strategies for gout in high-risk populations. We conducted a comprehensive meta-analysis integrating 52 global studies across four beverage categories using restricted cubic spline models. Compared with conventional categorical models, our continuous dose–response analyses revealed more granular, non-linear trends. Unlike prior reviews, this study simultaneously evaluated associations with two clinically distinct outcomes, hyperuricemia and gout, within a unified analytical framework, applied restricted cubic spline models to characterize non-linear dose–response relationships, and incorporated sex-stratified analyses to identify population-specific susceptibilities. This integrative approach—combining an updated evidence base, separate dual-outcome assessment, and continuous dose–response modeling across all four beverage categories—provides a more comprehensive foundation for dietary management and prevention of hyperuricemia and gout in high-risk populations.

## Materials and methods

### Search strategy

We performed an in-depth literature review utilizing major English academic databases, namely PubMed, Embase, the Cochrane Library, and Web of Science. The initial search encompassed the period starting from the creation of each database until December 20, 2025, and was subsequently updated, concluding on April 11, 2026. Refer to Supplementary methods 1 for the detailed search strategy. Additionally, to ensure comprehensive coverage, the bibliographies of the selected papers and related systematic reviews were manually examined for any relevant literature not captured during the primary database screening. The strategy involved the use of Medical Subject Headings (MeSH) and free-text terms, applying Boolean operators (OR/AND), alongside gray literature to gather supplementary information. Two reviewers, Mengxiao Li and Jiaying Wang, independently carried out the literature screening and data extraction processes. Discrepancies were settled by reaching a consensus or by seeking the opinion of a third reviewer, Wei Li. Initially, titles and abstracts were screened for relevance, and then a full-text review was conducted to determine eligibility based on set criteria. The review was systematically conducted following PRISMA guidelines and registered on PROSPERO (CRD420261279131).

### Inclusion and exclusion criteria

#### Inclusion criteria

The studies met the following conditions: (1) Researchers employed cross-sectional, cohort, or case–control designs in the study; (2) Participants included individuals with a confirmed diagnosis of HUA, typically defined by serum uric acid levels reaching or exceeding 7.0 mg/dL or 420 μmol/L in males, and 6.0 mg/dL or 360 μmol/L in females, or gout, diagnosed in accordance with the American College of Rheumatology (ACR) criteria or International Classification of Diseases (ICD) codes (11, 12); (3) The studies explicitly assessed the intake of specified beverages using categorical or continuous exposure metrics; (4) Relevant effect estimates, including ORs, relative risks (RRs), or HRs, were provided, accompanied by 95% CIs. A detailed explanation of technical terms is available in Supplementary Table S1.

#### Exclusion criteria

(1) Duplicate publications or reports involving overlapping study populations; (2) Articles lack full texts or essential extractable data unobtainable from the authors; (3) reviews, letters, conference abstracts, or case reports. Detailed PECOTS criteria are outlined in Supplementary Table S2.

### Literature screening and data extraction

Duplicate records were removed utilizing EndNote X9 software. A preliminary review of titles and abstracts was conducted, followed by a thorough evaluation of full texts to determine eligibility, with documented justifications for any exclusions. The data extracted encompassed the following variables: first author, year of publication, country, mean age, sample size, type of exposure and assessment method, data source, study design, disease classification, diagnostic criteria, and effect estimates with corresponding 95% CIs. In instances where only prevalence data were provided, case numbers were derived based on the total sample size. Effect estimates were directly extracted from the reports when raw data were not available. The authors were emailed to request any missing data.

### Quality assessment

The methodological quality of cross-sectional studies was evaluated using the Agency for Healthcare Research and Quality (AHRQ) scale, which categorizes scores into low (0–3), moderate (4–7), and high (8–11) quality levels (13). The Newcastle-Ottawa Scale (NOS) was used for case–control and cohort studies, with a score of 6 or higher signifying high quality (14).

### Statistical analysis

All statistical analyses were conducted utilizing Stata 18.0 software. We aggregated the extracted RRs and ORs. Where studies reported RRs rather than ORs, RR and OR were treated as numerically equivalent based on the rare-disease approximation: OR = RR × (1 − P₀) / (1 − RR × P₀) ≈ RR when the baseline incidence P₀ is small (15). However, the vast majority of included HUA studies reported ORs from cross-sectional or case–control designs, and the small proportion of cohort studies reporting RR were pooled (16). HRs were pooled separately and reported with their corresponding 95% CIs (17). The evaluation of statistical heterogeneity was conducted using the I^2^ statistic and Cochran’s Q test. When heterogeneity was low (I^2^ < 50.000% and *p* ≥ 0.100), a fixed-effects model was chosen; in other cases, a random-effects model was employed. Subgroup analyses and meta-regression were performed using covariates such as sex and types of various beverages to explore potential sources of significant heterogeneity (18).

For dose–response meta-analyses, data from studies offering at least three exposure categories were extracted for continuous variable analysis. Exposure units were standardized across beverages and outcomes, with coffee and sugar-sweetened beverage consumption reported in milliliters per day (mL/day) for both outcomes. The measurement of tea intake was in milliliters per day (mL/day) for gout and in terms of consumption occasions per week (occasions/week) for hyperuricemia. The amount of alcohol consumed was measured in grams of ethanol per day (g ethanol/day) for both outcomes. When conversion was necessary, we utilized the cup or serving size, the ethanol content per beverage, and the reporting period outlined in the original study or dietary questionnaire. The conversion to daily intake involved dividing weekly and monthly intake amounts by 7 and 30, respectively. Weekly consumption frequencies were calculated by multiplying daily and monthly frequencies by 7 and 7/30, respectively. Consumption occasions were not equated with cups or servings unless the specific amount consumed per event was clearly specified. Data that could not be accurately converted to the necessary unit were omitted from the corresponding dose–response model. In order to avoid bias in methodology, doses with different physical units were not merged into a single continuous variable. The original definitions of exposure, along with the volumes or ethanol contents per unit and conversion formulas for each study, can be found in Supplementary Table S3. For exposure categories with defined lower and upper limits, the average of these limits was used as the representative dose. The minimum value for the lowest open-ended category was established at zero, while the maximum open-ended category was given a dose 1.5 times the lower boundary of the highest open-ended category. Dose–response curves were strictly confined to the reported exposure ranges, avoiding any extrapolation. To assess potential nonlinear relationships, restricted cubic splines with 3 to 5 knots at predetermined percentiles were utilized. Dose–response relationships were estimated using restricted cubic spline models, and overall associations and non-linear departures were assessed with statistical tests. Subsequently, dose–response curves along with their 95% CIs were plotted to identify specific risk thresholds or inflection points (19). Egger’s test was used to evaluate publication bias, where a *p*-value below 0.050 suggested evidence of funnel plot asymmetry. Sensitivity analyses employing the leave-one-out method were used to evaluate the robustness of the pooled results. All statistical evaluations were carried out as two-tailed tests with a significance level set at α = 0.050 (20).

## Results

### Literature search and screening process

The comprehensive search yielded 24,550 articles. Removing 4,927 duplicates left 19,623 unique articles. Title and abstract screening excluded 19,250 irrelevant articles, leaving 373 for full-text review. During full-text assessment, articles were excluded for: inappropriate design (n = 129), mismatched exposures or outcomes (n = 98), missing extractable data (n = 84), and overlapping cohorts (n = 10). Ultimately, the meta-analysis incorporated 52 studies (21–72). Figure 1 displays the literature screening procedure.

**Figure 1:**
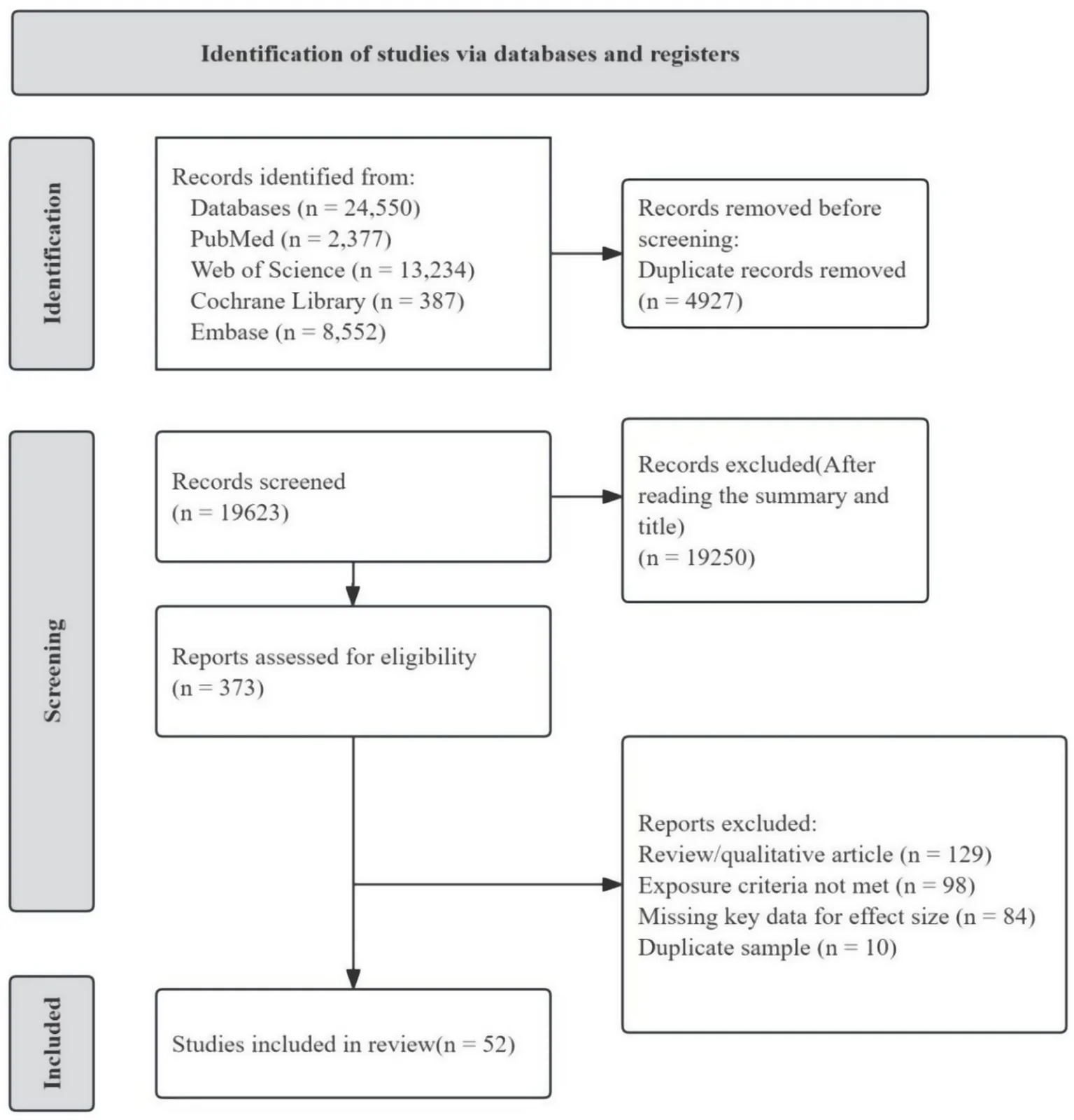
Flow diagram of study selection.

### Basic characteristics of included studies

Among the 52 studies incorporated, published between 2003 and 2024, there were 26 cross-sectional studies, 20 cohort studies, and 6 case–control studies. The research was conducted across various countries, including China, the United States, South Korea, the United Kingdom, Japan, Singapore, Brazil, Mexico, New Zealand, and several European nations. The beverage exposures evaluated encompassed coffee (21–30), alcohol (30–55), sugar-sweetened beverages (29, 30, 56–69), and tea (21–23, 25, 27, 29, 30, 50, 51, 70–72). The outcomes assessed included HUA (n = 31) and gout (n = 21). Detailed specifics of the study are provided in Supplementary Table S4.

### Quality assessment of included studies

Quality assessment using the AHRQ scale revealed that all 26 cross-sectional studies were of high quality. Points were primarily deducted because most cross-sectional surveys relied on self-reported data and lacked assessor blinding. Similarly, all 20 cohort studies achieved high-quality ratings based on the NOS scale. Minor deductions in this category were mainly due to unreported follow-up durations, or unclear attrition rates. Of the six case–control studies, four were deemed high-quality and two moderate-quality. Point deductions were largely attributed to the use of hospital-derived control groups and retrospective, questionnaire-based exposure assessments. Comprehensive quality assessment scores are detailed in Supplementary Tables S5–S7.

### Meta-analysis results

#### Risk of gout and hyperuricemia associated with coffee consumption

To explore the connection between coffee consumption and gout risk, the analysis incorporated three distinct studies: two cohort studies and a cross-sectional study. These studies encompassed a total of 266,268 participants. The heterogeneity test revealed significant variability among the studies (I^2^ = 80.200%, *p* = 0.006), requiring the application of a random-effects model for the combined analysis. The results suggested a potential reduction in gout risk among coffee consumers (OR = 0.543, 95% CI: 0.341–0.866, *p* = 0.010), as depicted in Figure 2A. However, given the substantial between-study heterogeneity (I^2^ = 80.2%), this estimate should be interpreted with caution, as it may reflect true variation in population characteristics, coffee types, or confounding profiles across studies rather than a uniform protective effect. The funnel plot and Egger’s test (Bias = −2.875, *p* = 0.310) both indicated no significant publication bias, with further details provided in Supplementary Figures S1, S2. The leave-one-out method was used for sensitivity analysis, and excluding a study from 2018 led to the lowest pooled odds ratio (OR = 0.426, 95% CI: 0.309–0.588). Further details on this analysis are provided in Supplementary Figure S3. Given that the 2018 study employed self-reported gout or the use of uric-acid-lowering medications as diagnostic criteria, encompassed over 130,000 European participants, and rigorously controlled for multiple dietary covariates and complications within the model, these elements likely contribute to the observed heterogeneity.

**Figure 2:**
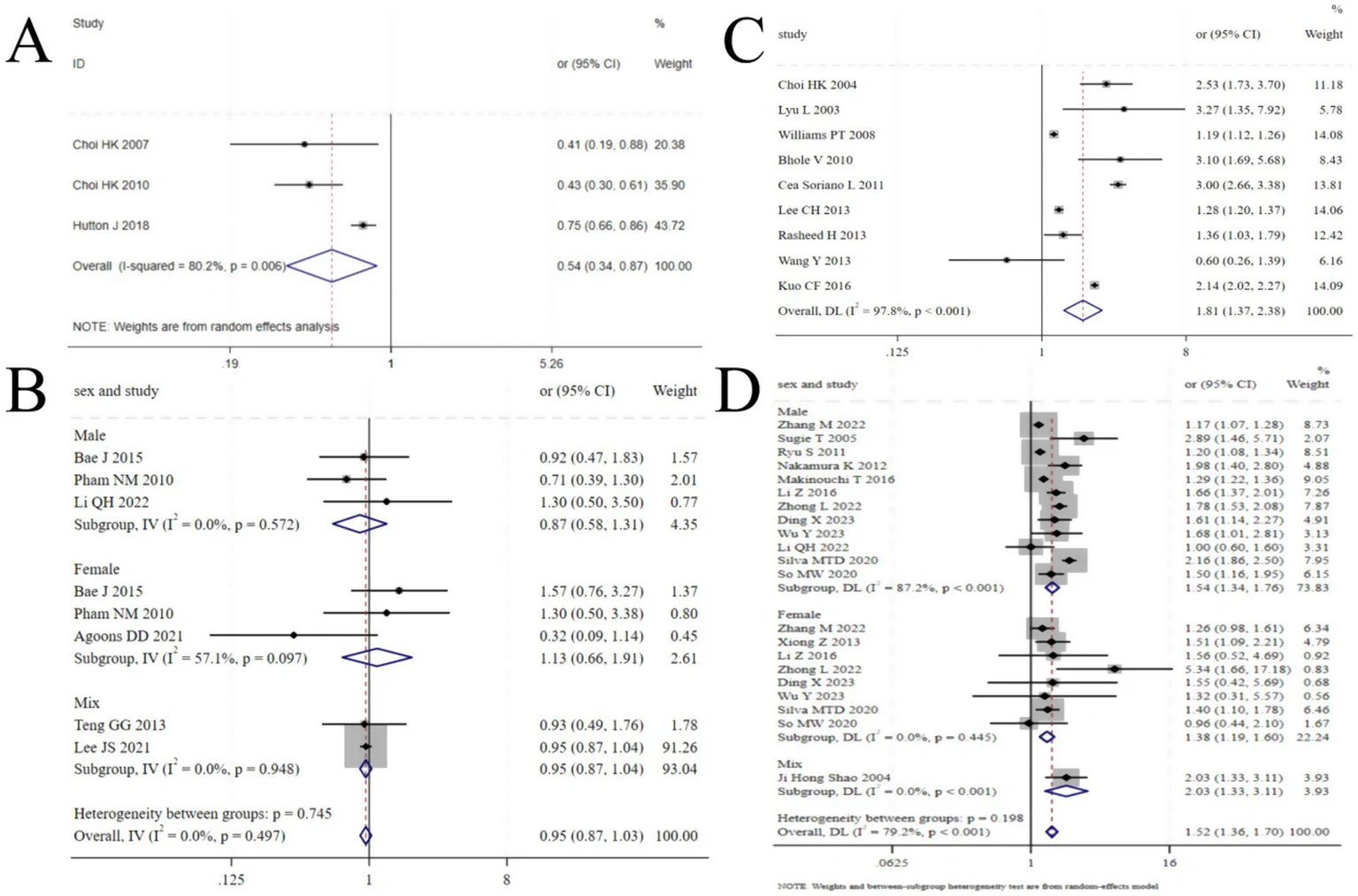
**(A)** Meta-analysis of the effect of coffee intake on gout. **(B)** Meta-analysis of the effect of coffee intake on hyperuricemia according to sex. **(C)** Meta-analysis of the effect of alcohol intake on gout. **(D)** Meta-analysis of the effect of alcohol intake on hyperuricemia according to sex.

In examining the relationship between coffee intake and HUA risk, a total of six independent studies were analyzed, including five cross-sectional studies and a cohort study, encompassing a sample size of 192,056 participants. No significant statistical heterogeneity was detected among the evaluated studies during the analysis (I^2^ < 0.001%, *p* = 0.497). Subsequent assessment utilizing a fixed-effects approach indicated that coffee intake was not significantly associated with HUA (OR = 0.950, 95% CI: 0.872–1.035, *p* = 0.240). Detailed results are presented in Figure 2B. The funnel plot and Egger’s test (Bias = −0.033, *p* = 0.945) both indicated no significant publication bias, with further details provided in Supplementary Figures S4, S5. Conducting a sensitivity analysis by systematically omitting individual studies showed that the pooled effect size varied minimally, and the direction of the results did not alter. This suggests that the direction of findings was generally consistent across sensitivity analyses, though some variability in effect size was observed, as depicted in Supplementary Figure S6.

To examine further the role of gender in the connection between coffee consumption and the development of HUA, a subgroup analysis stratified by gender was performed on studies that provided sex-specific data. Detailed findings are presented in Supplementary Figure S7. The analysis revealed that among male coffee consumers, there was a slight downward trend in the risk of developing HUA; nevertheless, this trend did not reach statistical significance (OR = 0.868, 95% CI: 0.577–1.306, *p* = 0.497), and there was no heterogeneity detected in this subgroup (I^2^ < 0.001%, *p* = 0.572). On the other hand, female coffee drinkers did not show a notable rise in risk (OR = 1.126, 95% CI: 0.665–1.909, *p* = 0.658), despite moderate heterogeneity in this group (I^2^ = 57.100%, *p* = 0.097). Furthermore, in the mixed-population subgroup, which did not distinguish by gender, no statistically significant association was observed (OR = 0.950, 95% CI: 0.869–1.037, *p* = 0.252), with no heterogeneity detected (I^2^ < 0.001%, *p* = 0.948). The test for between-group heterogeneity indicated that differences across gender subgroups did not reach statistical significance (*p* = 0.745). Overall, the aggregated results indicate that a consistent statistical relationship between coffee intake and the risk of developing HUA has not been definitively established. Notably, the opposing directional trends observed between male (OR = 0.868) and female (OR = 1.126) subgroups may reflect sex-based differences in uric acid metabolism, including the uricosuric effects of estrogen in premenopausal women, or variations in confounding exposures such as dietary patterns and comorbidities. Although between-subgroup differences did not reach significance (*p* = 0.745), these divergent trends warrant cautious interpretation and confirmation in future sex-stratified prospective studies.

#### Risk of gout and hyperuricemia associated with alcohol

Regarding the correlation between drinking alcohol and the likelihood of developing gout, this analysis encompassed nine independent studies, comprising a total of 273,282 participants. Testing for heterogeneity indicated substantial variance across the included research (I^2^ = 97.800%, *p* < 0.001); consequently, a random-effects framework was utilized for data integration. The pooled results established that alcohol intake was associated with higher odds of gout (OR = 1.809, 95% CI: 1.372–2.385, *p* < 0.001), with comprehensive data mapped out in Figure 2C. The funnel plot and Egger’s regression test (bias = 1.765, *p* = 0.636) both indicated no significant publication bias, with further details provided in Supplementary Figures S8, S9. Leave-one-out sensitivity analysis demonstrated that the pooled OR remained stable, ranging from 1.660 to 1.943 across all omission scenarios, with the highest estimate observed upon exclusion of Wang Y 2013 (OR = 1.943, 95% CI: 1.463–2.582) and the lowest upon exclusion of Cea Soriano L 2011 (OR = 1.660, 95% CI: 1.270–2.169). Specific results are presented in Supplementary Figure S10. In all omission analyses, the lower bounds of the 95% confidence intervals stayed above 1, confirming the strength of the positive association. Subgroup analysis by alcohol type, as presented in Supplementary Figure S11, indicated no statistically significant associations with gout for beer intake (OR = 1.328, 95% CI: 0.713–2.476, *p* = 0.371), spirits intake (OR = 1.250, 95% CI: 0.813–1.921, *p* = 0.309), and wine intake (OR = 1.495, 95% CI: 0.949–2.356, *p* = 0.083).

Meta-regression analyses were performed to explore potential sources of the substantial between-study heterogeneity observed for alcohol and gout risk (I^2^ = 97.800%). Three study-level covariates were examined in separate univariable meta-regression analyses: geographic region, sex composition, and study design. Detailed results are presented in Supplementary Table S8. None reached statistical significance. These results suggest that the high heterogeneity may reflect unmeasured factors such as alcohol type, quantity definition, and population-level variation in uric acid metabolism.

This study performed a distinct meta-analysis on studies that provided HR effect sizes to examine the time-dependent link between alcohol intake and the risk of developing gout during follow-up. The pooled analysis incorporated three cohort studies, utilizing HRs as the measure of effect. The analysis showed significant variability among the studies (I^2^ = 90.100%, *p* < 0.001), requiring the application of a random-effects model. The results showed that drinking alcohol greatly raised the hazard of developing gout (HR = 1.554, 95% CI: 1.237–1.953, *p* < 0.001). Specific results are presented in Supplementary Figure S12. The funnel plot and Egger’s test (Bias = −9.299, *p* = 0.349) both indicated no significant publication bias, as shown in Supplementary Figures S13, S14. However, given that only three datasets were included, potential publication bias cannot be completely ruled out. Leave-one-out sensitivity analysis showed consistent direction of association across all exclusion scenarios, as shown in Supplementary Figure S15.

In exploring the connection between drinking alcohol and the likelihood of HUA, this analysis incorporated data from 14 independent studies, encompassing a total of 456,997 participants. The heterogeneity test results indicated substantial statistical heterogeneity among the studies (I^2^ = 79.200%, *p* < 0.001), requiring the application of a random-effects model for the combined analysis. Given that some studies provided data stratified by gender, separate analyses were conducted for male, female, and mixed-gender groups. According to the random-effects meta-analysis, alcohol intake significantly heightened the risk of HUA (OR = 1.524, 95% CI: 1.363–1.703, *p* < 0.001), as illustrated in Figure 2D. The results indicate that consuming alcohol is a major risk factor for developing HUA among the general population. In males, alcohol use was notably connected to an increased risk of HUA (OR = 1.536, 95% CI: 1.342–1.757, *p* < 0.001), despite the presence of high heterogeneity (I^2^ = 88.200%, *p* < 0.001). The study found a notable link between alcohol consumption in women and a higher risk of occurrence (OR = 1.381, 95% CI: 1.191–1.601, *p* < 0.001), with no statistical heterogeneity observed within the female subgroup (I^2^ < 0.001%). The mixed-sex subgroup contained only one study, which reported a positive association (OR = 2.032, 95% CI: 1.327–3.111, *p* = 0.001); between-study heterogeneity could not be estimated for this subgroup. The test for subgroup differences was not statistically significant (*p* = 0.198). The funnel plot and Egger’s test (Bias = 1.310, *p* = 0.053) both indicated no significant publication bias, with further details provided in Supplementary Figures S16, S17. Sensitivity analysis indicated that excluding the 2020 study yielded the lowest pooled odds ratio (OR = 1.456, 95% CI: 1.323–1.601). Specific results are presented in Supplementary Figure S18. Separate subgroup analyses by type of alcohol were performed for men and women. In males, there was a significant positive relationship between spirits intake and hyperuricemia (OR = 1.320, 95% CI: 1.141–1.527, *p* < 0.001), while beer intake (OR = 1.260, 95% CI: 0.990–1.604, *p* = 0.060) and wine intake (OR = 1.088, 95% CI: 0.934–1.267, *p* = 0.277) had no significant association. In women, beer intake (OR = 1.320, 95% CI: 0.966–1.803, *p* = 0.081), wine intake (OR = 0.805, 95% CI: 0.599–1.083, *p* = 0.153), and spirits intake (OR = 1.319, 95% CI: 0.733–2.371, *p* = 0.356) all showed no significant association with hyperuricemia. Specific results for these subgroups are seen in Supplementary Figures S19, S20.

#### Risk of gout and hyperuricemia associated with sugar-sweetened beverages

While investigating the connection between the intake of sugar-sweetened beverages and gout risk, this research incorporated data from four independent studies, encompassing a total of 146,878 participants. The heterogeneity test indicated moderate statistical heterogeneity among the studies (I^2^ = 68.700%, *p* = 0.023), requiring the application of a random-effects model for the combined analysis. The study showed that drinking sugar-sweetened beverages was linked to a higher risk of gout compared to those with little or no consumption (OR = 1.371, 95% CI: 1.059–1.776, *p* = 0.017), as detailed in Figure 3A. The funnel plot and Egger’s test (*p* = 0.089) are presented in Supplementary Figures S21, S22. In the sensitivity analysis, after using the leave-one-out method to exclude the 2014 study, the pooled OR of the association between SSB intake and gout increased significantly, yielding the highest value (OR = 1.639, 95% CI: 1.059–2.539). This variation may be due to the bias caused by subjects in this cross-sectional study changing their lifestyles after receiving gout health education. Nevertheless, the direction of the association remained positive, but statistical significance was not maintained in all leave-one-out analyses. Specific results are presented in Supplementary Figure S23.

**Figure 3:**
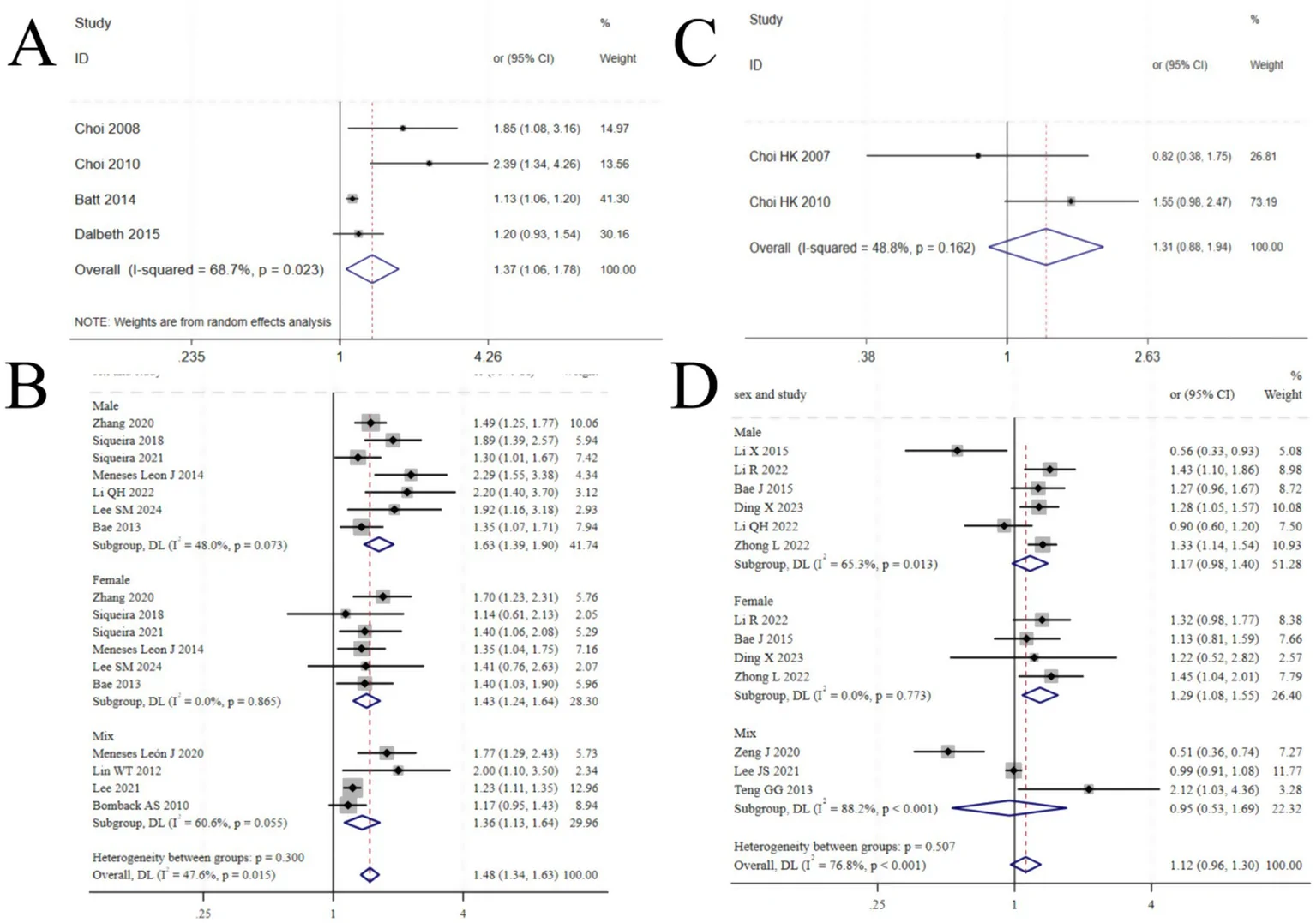
**(A)** Meta-analysis of the effect of SSB intake on gout. **(B)** Meta-analysis of the effect of SSB intake on hyperuricemia according to sex. **(C)** Meta-analysis of the effect of tea intake on gout. **(D)** Meta-analysis of the effect of tea intake on hyperuricemia according to sex.

Exploring the connection between the consumption of sugar-sweetened beverages and the potential risk of HUA, this study incorporated data from 11 studies, encompassing a total of 249,882 participants. The heterogeneity test results indicated substantial statistical heterogeneity among the studies (I^2^ = 47.600%, *p* = 0.015), requiring the application of a random-effects model. Research indicated that SSB consumption was associated with higher odds of HUA (OR = 1.478, 95% CI: 1.342–1.627, *p* < 0.001), as depicted in Figure 3B. Specifically, within the male subgroup, which included seven studies, consuming sugar-sweetened drinks was linked to a notably higher risk of HUA (OR = 1.627, 95% CI: 1.390–1.903, *p* < 0.001). The intake of sugar-sweetened beverages by women similarly raised the likelihood of developing HUA (OR = 1.426, 95% CI: 1.237–1.644, *p* < 0.001). Egger’s test revealed asymmetry in the funnel plot (*p* = 0.004), suggesting potential small-study effects such as publication bias, as detailed in Supplementary Figures S24, S25. In sensitivity analyses where one estimate was left out, the combined ORs varied from 1.435 to 1.511, with all related 95% confidence intervals excluding 1. Further specific results are available in Supplementary Figure S26.

#### Risk of gout and hyperuricemia associated with tea consumption

Concerning the link between drinking tea and the likelihood of developing gout, this analysis incorporated data from only two independent studies, encompassing a total of 135,302 participants. The heterogeneity test indicated moderate heterogeneity between these studies (I^2^ = 48.800%, *p* = 0.162), resulting in the use of a fixed-effects model. The study found no meaningful link between drinking tea and the likelihood of getting gout (OR = 1.307, 95% CI: 0.880–1.940, *p* = 0.185), as detailed in Figure 3C. Given the currently limited evidence and the fact that the studies focused on male and female populations separately, tea consumption cannot yet be classified as an independent risk or protective factor for gout incidence. Owing to the inclusion of only two studies in this analysis, additional high-quality, large-scale original research is urgently needed to supply more solid evidence moving forward.

With respect to the connection between tea drinking and HUA risk, this analysis incorporated data from nine independent studies, encompassing a total of 222,383 participants. A substantial amount of statistical heterogeneity among the studies was shown by the heterogeneity test (I^2^ = 76.800%, *p* < 0.001), necessitating the use of a random-effects model. Tea intake did not show a notable connection with the likelihood of developing hyperuricemia (OR = 1.119, 95% CI: 0.961–1.304, *p* = 0.147), with detailed results presented in Figure 3D. In subgroup analyses stratified by gender, the female subgroup suggested a significant increase in hyperuricemia risk associated with tea consumption (OR = 1.294, 95% CI: 1.081–1.549, *p* = 0.005), but tea consumption was not significantly associated with HUA in the male subgroup (OR = 1.169, 95% CI: 0.977–1.399, *p* = 0.089). Conversely, there was no significant link between tea consumption and hyperuricemia risk in the subgroup that included both male and female participants (OR = 0.948, 95% CI: 0.532–1.689, *p* = 0.857). The funnel plot and Egger’s test both did not provide statistically significant evidence of funnel plot asymmetry, with further details provided in Supplementary Figures S27, S28. Sensitivity analysis revealed that excluding a study from 2020 resulted in the highest pooled odds ratio (OR = 1.191, 95% CI: 1.042–1.361), with specific findings presented in Supplementary Figure S29. A literature review identified that these data were derived from a univariate logistic regression model lacking adjustments for critical confounding variables, including gender, BMI, alcohol consumption, and high-purine diet. Due to potential confounding, the unadjusted estimate should be considered with caution.

Meta-regression analyses were performed to explore potential sources of the substantial between-study heterogeneity observed for tea and HUA risk (I^2^ = 76.8%). Four study-level covariates were examined individually: tea type, geographic region, study design, and sex composition. Detailed results are presented in Supplementary Table S9. None of the moderators analyzed reached statistical significance, but the small number of studies limited the ability to detect sources of heterogeneity, and the variability observed might be due to unmeasured factors such as tea preparation method or population-level variation in uric acid metabolism.

### Dose–response analysis of different beverage intakes and the risk of gout and hyperuricemia

For studies that reported multiple exposure categories, we further conducted a dose–response analysis. Depending on the measurement methods used in the included studies, the relationships between different beverages and the risk of gout were fitted using the following units: daily coffee intake (mL/day), daily alcohol intake (g ethanol/day), daily SSB intake (mL/day), and daily tea intake (mL/day). For hyperuricemia, dose–response relationships were modeled using daily coffee intake (mL/day), daily alcohol intake (g ethanol/day), daily SSB intake (mL/day), and tea consumption frequency (occasions/week).

#### Dose–response analysis of coffee intake and risk of gout and hyperuricemia

In relation to daily coffee intake (mL/day) and gout risk, two prospective cohorts encompassing 135,302 participants were included. There was an inverse relationship between daily coffee intake (mL/day) and the risk of developing gout, as suggested by the dose–response analysis, with no nonlinear patterns detected (*p* = 0.341), which is detailed in Figure 4A and Supplementary Table S10. The link between coffee intake and the risk of developing gout was statistically significant (*p* < 0.001). Observations from the fitted curve indicated a negative correlation when coffee intake ranged from 0 to 2,133 mL/day, with the risk consistently decreasing as intake increased. Specifically, compared to non-consumers (0 mL/day), consuming 592.5 mL/day decreased the odds (OR = 0.820, 95% CI: 0.710–0.940); consuming 1066.5 mL/day decreased the odds (OR = 0.560, 95% CI: 0.440–0.710); and consuming 1,422 mL/day decreased the odds (OR = 0.450, 95% CI: 0.340–0.600). At the highest analyzed intake of 2,133 mL/day, the estimated association remained inverse (OR = 0.380, 95% CI: 0.190–0.740), though this estimate should be interpreted with caution. The connection between daily coffee consumption (mL/day) and the risk of HUA was investigated through a cross-sectional study that included 11,662 participants. According to the dose–response analysis, there was no statistically significant connection between coffee consumption and hyperuricemia risk (*p* = 0.118), nor was there a significant non-linear relationship (*p* = 0.262). Figure 4B and Supplementary Table S11 provide a detailed illustration of the findings. The overall association was not statistically significant, and the findings require confirmation in prospective studies. With regard to daily coffee intake (mL/day) and HUA risk in males, one cross-sectional study encompassing 4,964 participants was included. The analysis of dose–response did not provide statistically significant evidence of an overall association between daily coffee intake and the risk of hyperuricemia in men (*p* = 0.051), even though the non-linear test reached statistical significance (*p* = 0.044). Supplementary Figure S30 and Supplementary Table S12 display these findings. In contrast, according to the same cross-sectional study, which included 6,698 female participants, there was no significant statistical relationship between women’s daily coffee consumption and their risk of hyperuricemia (*p* = 0.904), and no significant non-linear relationship was found either (*p* = 0.771). Supplementary Figure S31 and Supplementary Table S13 contain detailed results.

**Figure 4:**
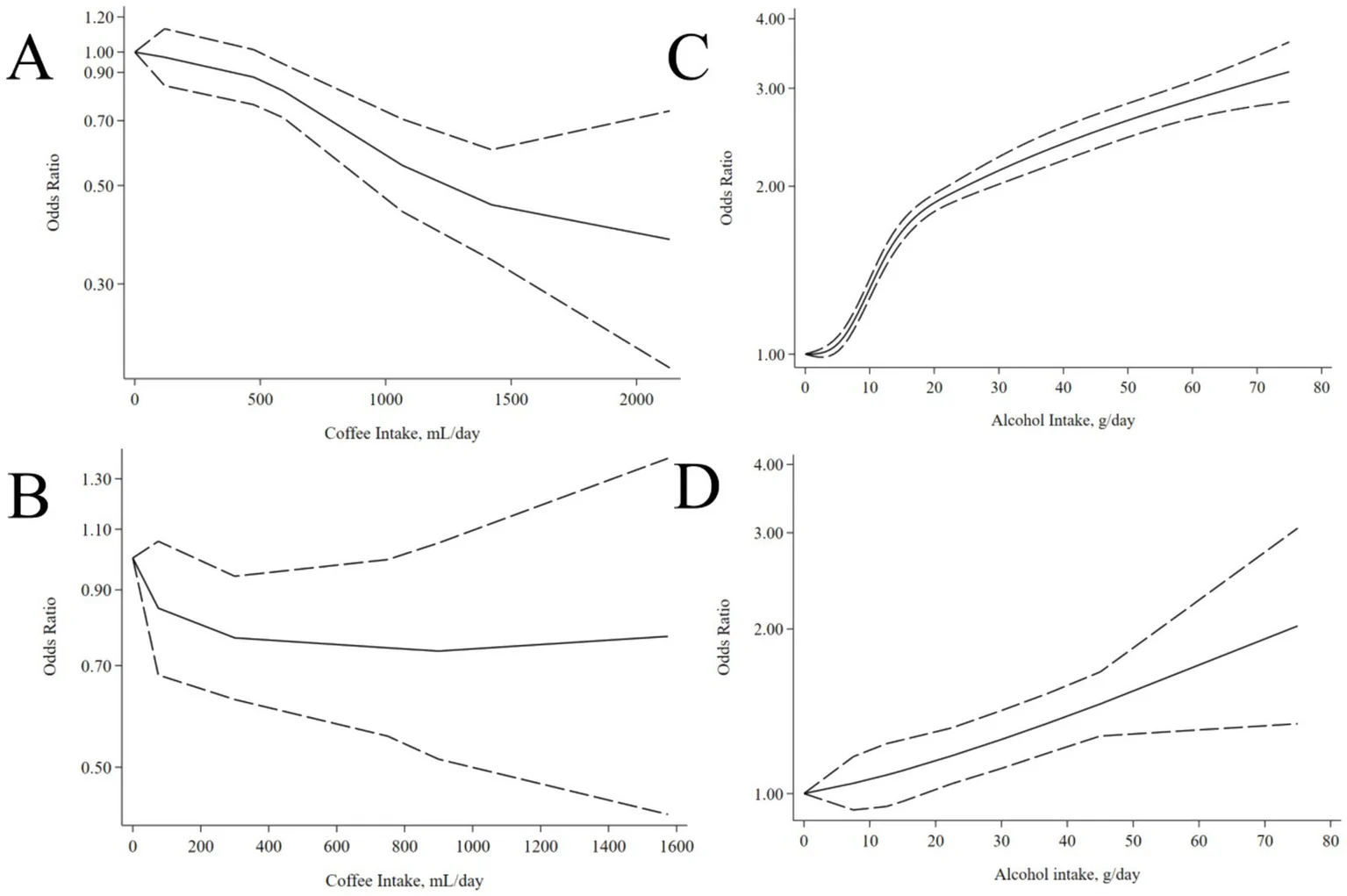
Dose–response analysis of the association between various beverage intakes and the risk of gout and hyperuricemia. **(A,B)** Coffee intake (mL/day) and the risk of gout **(A)** and hyperuricemia **(B)**. **(C,D)** Alcohol intake (g/day) and the risk of gout **(C)** and hyperuricemia **(D)**.

#### Dose–response analysis of alcohol intake and risk of gout and hyperuricemia

To assess the relationship between daily alcohol consumption and gout risk, the analysis utilized data from two case–control studies and one cohort study, collectively involving 186,305 participants. The analysis of dose–response showed a statistically significant association between alcohol consumption and the likelihood of developing gout (*p* < 0.001), demonstrating a notable non-linear dose–response relationship (*p* < 0.001). The comprehensive results can be found in Figure 4C and Supplementary Table S14. The dose–response curve fit indicated a nonlinear positive relationship between daily alcohol consumption (g ethanol/day) and the risk of gout, where increased intake was linked to higher estimated odds of developing gout.

With regard to daily alcohol intake (g ethanol/day) and HUA risk, one prospective cohort study and two cross-sectional studies encompassing 22,369 participants were included. The analysis of dose–response revealed a positive association, with no statistical evidence of nonlinearity between daily alcohol consumption (g ethanol/day) and the likelihood of developing hyperuricemia, with an overall trend *p*-value of less than 0.001 and a non-linear trend *p*-value of 0.820. These findings are elaborated in Figure 4D and Supplementary Table S15. When alcohol intake ranged from 0 to 75 g ethanol/day, the risk of hyperuricemia was positively correlated with alcohol consumption, increasing as intake increased. One prospective cohort study and two cross-sectional studies, which included a total of 16,327 male participants, were analyzed to examine the link between daily alcohol consumption (g ethanol/day) and hyperuricemia risk. The dose–response study identified a significant link between men’s daily alcohol consumption and hyperuricemia risk (*P* < 0.001), but the non-linear trend was not significant (*p* = 0.817). Supplementary Figure S32 and Supplementary Table S16 contain detailed findings. When alcohol intake ranged from 0 to 75 g ethanol/day, men’s hyperuricemia risk was positively correlated with alcohol consumption.

#### Dose–response analysis of SSB intake and risk of gout and hyperuricemia

Two prospective cohort studies, which included 125,299 participants, examined the link between daily consumption of SSBs (mL/day) and the likelihood of developing gout. The analysis of dose–response showed a notable linear relationship between daily consumption of SSBs and the risk of gout (overall trend *p* < 0.001; non-linear trend *p* = 0.162). Detailed findings are illustrated in Figure 5A and Supplementary Table S17. When SSB intake ranged from 278.93 to 1,065 mL/day, gout risk was positively correlated with intake. The overall trend showed a positive association, suggesting that higher daily SSB intake may be associated with an elevated risk of gout across the observed intake range. In relation to daily SSB intake (mL/day) and gout risk in males, one prospective cohort study encompassing 46,393 participants was included. The analysis of dose–response showed a statistically significant link between SSB consumption and gout risk in men (*p* = 0.033), although no significant non-linear relationship was found (*p* = 0.300). Detailed findings are presented in Supplementary Figure S33 and Supplementary Table S18. When SSB intake ranged from 278.93 to 1,065 mL/day, men’s gout risk was positively correlated with intake. Because this subgroup analysis only included a single study, these results require further validation from high-quality cohort studies. Turning to daily SSB intake (mL/day) and gout risk in females, one prospective cohort study encompassing 78,906 participants was included. The dose–response study revealed a statistically significant connection between SSB intake and the risk of gout in women (*p* = 0.001), with a significant non-linear association (*p* = 0.033). Detailed findings can be found in Supplementary Figure S34 and Supplementary Table S19. The fitted curve showed that when SSB intake ranged from 355 to 1,065 mL/day, women’s gout risk was positively correlated with intake. The overall trend suggests that higher daily SSB intake may also be associated with an increased risk of gout in women. This dose–response analysis was based on a single cohort study and should be interpreted as exploratory; the curve essentially reproduces the exposure-outcome gradient of that one study and does not constitute a pooled estimate. In relation to daily SSB intake (mL/day) and HUA risk, one cross-sectional study and one prospective cohort study encompassing 8,005 participants were included. The analysis of dose–response showed a notable nonlinear correlation between the daily consumption of SSB (mL/day) and the risk of hyperuricemia, with an overall trend *p*-value of less than 0.001 and evidence of nonlinearity (*p* = 0.003). Within the intake range of 0–1597.5 mL/day, the estimated likelihood of HUA generally rose with SSB consumption, though these results are exploratory due to the limited number of studies involved. These results are illustrated in Figure 5B and Supplementary Table S20.

**Figure 5:**
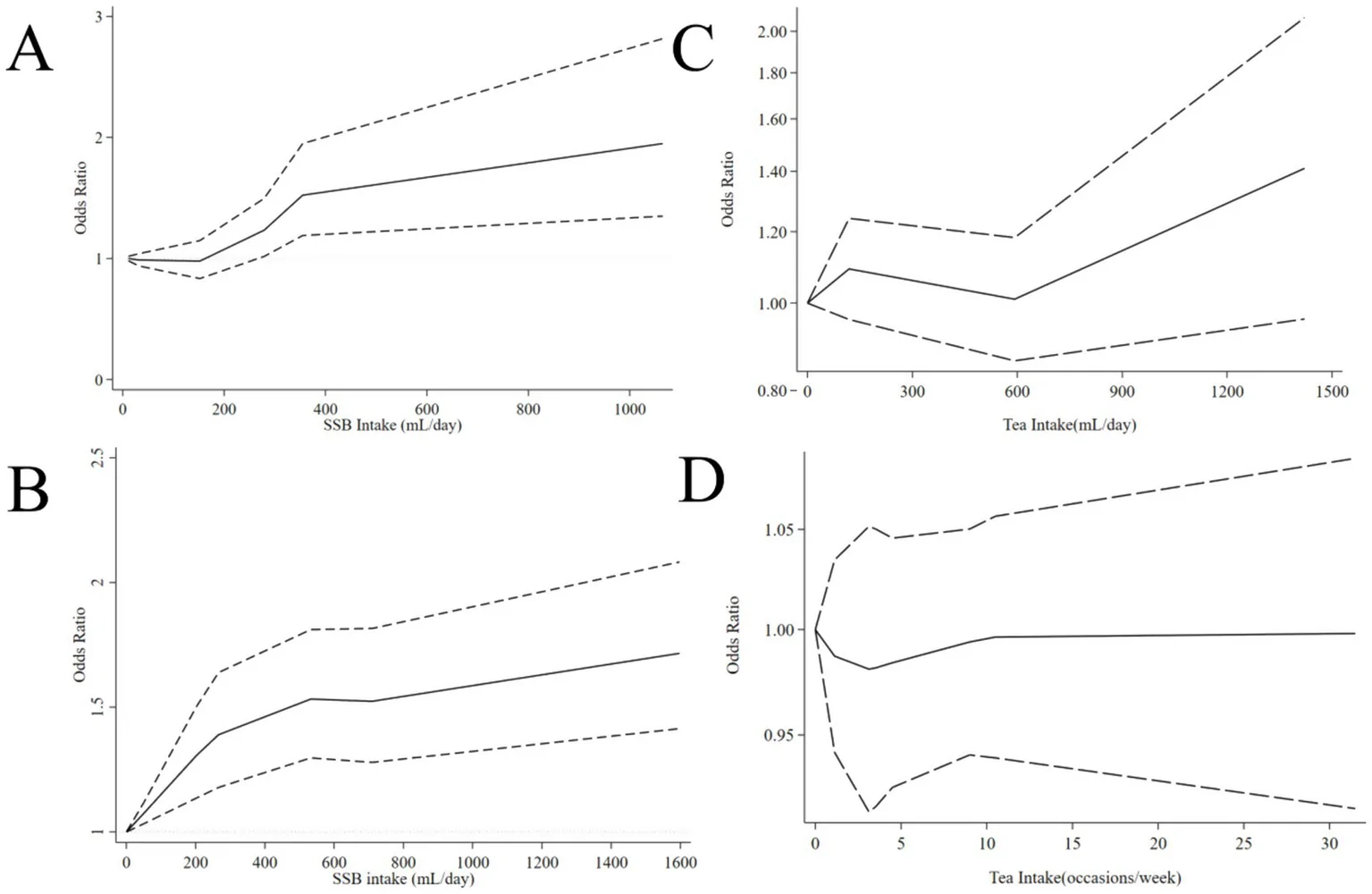
Dose–response analysis of the association between various beverage intakes and the risk of gout and hyperuricemia. **(A,B)** Sugar-sweetened beverage intake (mL/day) and the risk of gout **(A)** and hyperuricemia **(B)**. **(C,D)** Tea intake and the risk of gout (mL/day) **(C)** and hyperuricemia (occasions/week) **(D)**.

#### Dose–response analysis of tea intake and risk of gout and hyperuricemia

Two prospective cohort studies with a combined total of 135,302 participants were examined to assess the link between daily tea consumption (mL/day) and the risk of gout. The dose–response evaluation did not provide evidence of a statistically significant association with gout (*p* = 0.210), alongside showing no significant non-linear association (*p* = 0.127). Specific results for this analysis are found in Figure 5C and Supplementary Table S21. The fitted curve indicated that higher daily tea intake might increase the risk of gout; however, because this analysis only included two studies and featured limited dosage strata, these findings necessitate further validation from high-quality research. Three studies were included in a dose–response meta-analysis of tea consumption frequency (occasions/week) and HUA. Restricted cubic spline analysis showed no significant overall association (*p* = 0.960) or evidence of nonlinearity (*p* = 0.860). The fitted curve and detailed estimates are presented in Figure 5D and Supplementary Table S22.

## Discussion

### Summary of Main findings

The current study investigated the epidemiological associations and dose–response relationships between the consumption of coffee, alcohol, sugar-sweetened beverages, and tea, and the risk of HUA and gout. The findings indicated that coffee consumption was associated with a reduced risk of gout, whereas the intake of alcohol and sugar-sweetened beverages was linked to an increased risk of both gout and HUA. Subgroup analyses revealed that alcohol consumption elevated the risk of hyperuricemia across genders, with spirits showing a significant positive association in males. Additionally, tea consumption was associated with an increased risk of hyperuricemia in female subgroups. The analysis of dose–response data indicated a negative correlation between coffee consumption and gout, with no statistical evidence of a nonlinear pattern. On the other hand, drinking alcohol showed a positive dose–response relationship with both gout and HUA, with gout displaying signs of nonlinearity. Likewise, the consumption of SSBs was positively associated with both conditions, showing a nonlinear relationship with HUA in the overall analyses. There was no statistically significant dose–response relationship found between coffee intake and HUA, nor between tea intake and either condition. The results should be viewed carefully because of the observational study designs, significant variability in several analyses, and the small number of studies involved in the dose–response analyses.

### Comparison with previous meta-analyses

This review builds on earlier meta-analyses by integrating evidence on coffee, tea, alcohol, and sugar-sweetened beverage consumption in relation to two distinct outcomes: gout and hyperuricemia. Although recent meta-analyses have examined these outcomes and conducted subgroup analyses within specific beverage categories, our study brings these previously separate bodies of evidence into a common analytical framework. We supplemented categorical comparisons with outcome-specific dose–response analyses where sufficient data were available. This approach provides a broader assessment of the direction and patterns of associations across intake levels while maintaining a clear distinction between hyperuricemia and clinically manifest gout. The principal analytical contribution of this review lies in the characterization of outcome-specific dose–response relationships. Whereas a systematic review published in 2025 examined drinking frequency (73), we expressed alcohol exposure in grams of ethanol per day and analyzed its association with each outcome separately. This distinction is important because drinking frequency does not necessarily reflect the amount of ethanol consumed. Our analyses identified evidence of a nonlinear association with gout, whereas no statistically significant departure from linearity was detected for hyperuricemia. For sugar-sweetened beverages, we extended the categorical synthesis reported in a meta-analysis published in 2025 to examine associations across intake levels (74), identifying evidence of nonlinearity for hyperuricemia but not for gout. These analyses provide additional information on how associations vary with consumption, beyond determining whether higher intake is associated with either outcome. However, the limited dose–response evidence does not support definitive intake thresholds. Our findings also refine the interpretation of existing evidence. Consistent with the outcome-specific findings of a meta-analysis published in 2025 (75), coffee consumption was inversely associated with gout but showed no clear association with hyperuricemia. Our additional dose–response analysis supported the inverse association with gout without statistically significant evidence of nonlinearity. These findings reinforce the importance of avoiding a general claim that coffee consumption is associated with a lower occurrence of both conditions. We also handled hazard ratios separately from the other effect estimates, pooling them when feasible and otherwise reporting them narratively. Together, the broader coverage of beverage categories, outcome-specific dose–response analyses, and explicit handling of effect measures provide a more integrated account of the available evidence. These contributions aid the interpretation of observed associations, although the conclusions remain subject to the limitations of heterogeneous observational studies.

### Potential biological mechanisms

The protective benefits of coffee emerge from the combined action of its various bioactive compounds rather than a single ingredient. Polyphenols like chlorogenic acid play a central role in this process. Pharmacological evidence demonstrates that chlorogenic acid greatly enhances insulin sensitivity in target organs. From a physiological standpoint, hyperinsulinemia upregulates renal urate transporters such as URAT1 in the proximal convoluted tubule, which promotes the reabsorption of uric acid (1). Antioxidant compounds in coffee reduce insulin resistance and indirectly decrease this reabsorption, leading to improved uric acid excretion. The metabolites of caffeine, a methylxanthine derivative, competitively inhibit xanthine oxidase and delay the conversion of hypoxanthine to uric acid. Clinical crossover trials have also shown that caffeine boosts the renal clearance of uric acid (76). Alongside these metabolic changes, Mendelian randomization research indicates that the benefits of coffee go beyond simply lowering serum uric acid levels. Diterpenes like cafestol suppress the NLRP3 inflammasome pathway, providing an independent anti-inflammatory defense against gout (77). The suggested mechanisms might clarify the negative correlation between coffee intake and gout found in this analysis, although the dose–response analysis did not show statistically significant nonlinearity.

Alcohol serves as a confirmed risk factor for gout through biological mechanisms that simultaneously drive uric acid production and impair renal clearance. Hepatic ethanol metabolism severely depletes intracellular adenosine triphosphate by rapidly converting it into adenosine monophosphate. This accumulation of free adenosine monophosphate acts as a direct substrate for purine degradation and triggers a sharp surge in endogenous uric acid generation (78). The breakdown of alcohol also elevates circulating lactic acid levels. Lactic acid competes directly with uric acid for organic anion transporters during the process of renal excretion. Elevated lactic acid concentrations effectively block uric acid clearance and lead to widespread retention in the bloodstream (79). Alcohol consumption further disrupts the gut microbiome, which compromises the compensatory uric acid excretion capabilities of the intestines. These mechanisms provide biological plausibility for the positive association between alcohol intake and hyperuricemia observed in our analysis.

The harmful influence of SSBs on uric acid metabolism stems primarily from high concentrations of added fructose or high-fructose corn syrup. Fructose metabolism bypasses the standard negative feedback regulation of phosphofructokinase. Upon entering hepatocytes, fructokinase rapidly phosphorylates fructose through a mechanism that unrestrictedly consumes adenosine triphosphate. This rapid consumption leads to inorganic phosphate depletion and a sudden accumulation of adenosine monophosphate. Following a pathway similar to ethanol metabolism, this excess adenosine monophosphate degrades rapidly into uric acid. Chronic intake of sugar-sweetened beverages provokes systemic metabolic dysfunction alongside this acute uric acid generation. Nutritional evidence confirms that fructose actively promotes hepatic lipogenesis, which initiates severe chronic insulin resistance and impairs intestinal barrier function. These cascading effects progressively diminish the natural capacity of the body to excrete uric acid (80). The present dose–response analysis showed a positive nonlinear association between SSB intake and HUA. Metabolic evaluations show that higher circulating estrogen levels in women enhance endothelial function and directly facilitate renal tubular uric acid excretion. Estrogen-related differences in urate handling may be relevant to potential sex differences in this association. However, SSB intake was positively associated with HUA in both men and women, and our findings do not establish an estrogen-mediated protective effect (81).

Tea is generally considered beneficial due to its rich polyphenol content and antioxidant effects. Although tea consumption was not significantly associated with hyperuricemia overall, a positive association was observed in the female subgroup. The result is consistent with observations from cross-sectional studies in older Chinese adults, which show increased serum urate levels among those who habitually consume tea (82). This connection is probably facilitated by multiple interrelated biological processes. Firstly, the high levels of caffeine and theophylline in tea have significant diuretic effects, which, if consumed over time, might cause slight volume depletion and trigger the renin-angiotensin system. The activation leads to an increase in sodium reabsorption in the proximal tubule and concurrently raises uric acid reabsorption through transporters like URAT1. In addition, polyphenol metabolites, especially phenolic acids, serve as organic anions that could compete with uric acid for renal organic anion transporters during tubular secretion, potentially disrupting the usual urinary excretion of uric acid. Thirdly, the high oxalate content in tea may interfere with uric acid elimination at the excretory level, while tannins and other constituents can alter gut microbiota composition by inhibiting the growth of beneficial bacteria capable of degrading uric acid, a potentially vital pathway considering the recognized influence of intestinal microecology on the onset of hyperuricemia (83).

### Limitations

While this study provides large-scale epidemiological evidence, several limitations must be acknowledged. First, all included studies were observational. Although many studies adjusted for potential confounders, the covariates included in the models varied across studies. Nevertheless, beverage consumption remains deeply interconnected with broader dietary patterns like red meat or seafood intake and complex lifestyle habits, including physical activity. The nature of the original data prevents the complete elimination of residual confounding from the pooled effects. A scarcity of eligible literature severely restricted the statistical power within specific subgroup evaluations. For example, only two studies contributed to the pooled OR analysis of tea consumption and gout. With so few studies, funnel plots and Egger’s test cannot reliably assess small-study effects or exclude publication bias. Consequently, these subgroup conclusions remain highly vulnerable to the outsized influence of individual datasets and require extremely cautious interpretation. The comparability of exposure estimates could be limited by differences in serving definitions and dietary assessment methods used in various studies. Even though units were standardized based on data from the original studies, the converted values might not accurately reflect the same levels of actual consumption. The analysis of hyperuricemia measured tea consumption by frequency, preventing volume-based interpretation, potentially affecting dose–response curves and associations. These dose–response analyses are exploratory and should be approached with caution. The standard reliance on self-reported dietary intake through food frequency questionnaires introduces an unavoidable susceptibility to recall bias and exposure misclassification.

## Conclusion

In summary, coffee, tea, alcohol, and sugar-sweetened beverages showed different patterns of association with hyperuricemia and gout. Alcohol and sugar-sweetened beverage intake was positively associated with both outcomes, with dose–response analyses providing further support for these associations. Coffee consumption was inversely associated with gout, without statistically significant evidence of nonlinearity, but showed no clear association with hyperuricemia. Overall, tea consumption was not significantly associated with either outcome. Although tea consumption was positively associated with hyperuricemia in women, the difference between sexes was not statistically significant. In particular, the dose–response findings for sugar-sweetened beverage intake and gout in women were based on a single cohort study and require independent replication. This review could inform future research on beverage consumption and the prevention of hyperuricemia and gout. Large prospective cohort studies with clearly defined beverage types, standardized exposure assessment, and more comprehensive adjustment for dietary and lifestyle confounders are needed to clarify these associations.

## Data Availability

The original contributions presented in the study are included in the article/Supplementary material, further inquiries can be directed to the corresponding author.
