# Supplementary material for: Association between intake of different beverages and risk of hyperuricemia and gout: a risk factor and dose–response meta–analysis
Source: Front Nutr. 2026 Oct 22;13:1956907. doi: 10.3389/fnut.2026.1956907 (PMC13760532; doi:10.3389/fnut.2026.1956907)
Supplement: Supplementary file 1 [file Supplementary_file_1.docx]

**SUPPLEMENTARY MATERIAL**

**Table of Contents**

**Supplementary Methods 1 –** Database search strategy.

**Supplementary Table S1 –** Explanation of Statistical Terms and Abbreviations.

**Supplementary Table S2** – Population, Exposure, Comparator, Outcome, Time frame, Study design (PECOTS) approach.

**Supplementary Table S3** – Exposure unit conversion for dose-response analysis.

**Supplementary Table S****4 –** Demographic characteristics of included studies.

**Supplementary Table S5 –** Agency for Healthcare Research and Quality (AHRQ) assessment of the quality of cross-sectional studies.

**Supplementary Table S6 –** Newcastle-Ottawa Scale (NOS) Assessment of Cohort Study Quality.

**Supplementary Table S7 –** Newcastle-Ottawa Scale (NOS) Assessment of Case-control studies.

**Supplementary Figure S1 –** Funnel chart of coffee intake and gout risk.

**Supplementary Figure S2 –** Egger’s test of coffee intake and gout risk.

**Supplementary Figure S3 –** Sensitivity analysis of coffee intake and gout risk.

**Supplementary Figure S4 –** Funnel chart of coffee intake and hyperuricemia risk.

**Supplementary Figure S5 –** Egger’s test of coffee intake and hyperuricemia risk.

**Supplementary Figure S6 –** Sensitivity analysis of coffee intake and hyperuricemia risk.

**Supplementary Figure S7 –** Gender subgroup analysis examines the relationship between coffee intake and hyperuricemia risk.

**Supplementary Figure S8 –** Funnel chart of alcohol intake and gout risk.

**Supplementary Figure S9 –** Egger’s test of alcohol intake and gout risk.

**Supplementary Figure S10 –** Sensitivity analysis of alcohol intake and gout risk.

**Supplementary Figure S11 – The alcohol** categories subgroup analysis examines the relationship between alcohol intake and gout risk.

**Supplementary Table S8 –** Meta-regression results for alcohol consumption and gout risk.

**Supplementary Figure S12 –** Forest plot of alcohol intake and gout risk (HR).

**Supplementary Figure S13 –** Funnel chart of alcohol intake and gout risk (HR).

**Supplementary Figure S14 –** Egger’s test of alcohol intake and gout risk (HR).

**Supplementary Figure S15 –** Sensitivity analysis of alcohol intake and gout risk (HR).

**Supplementary Figure S16 –** Funnel chart of alcohol intake and hyperuricemia risk.

**Supplementary Figure S17 –** Egger’s test of alcohol intake and hyperuricemia risk.

**Supplementary Figure S18 –** Sensitivity analysis of alcohol intake and hyperuricemia risk.

**Supplementary Figure S19 –** Alcohol categories subgroup analysis in males examines the relationship between alcohol intake and hyperuricemia risk.

**Supplementary Figure S20 –** Alcohol categories subgroup analysis in females examines the relationship between alcohol intake and hyperuricemia risk.

**Supplementary Figure S21 –** Funnel chart of sugar-sweetened beverage intake and gout risk.

**Supplementary Figure S22 –** Egger’s test of sugar-sweetened beverage intake and gout risk.

**Supplementary Figure S23 –** Sensitivity analysis of sugar-sweetened beverage intake and gout risk.

**Supplementary Figure S24 –** Funnel chart of sugar-sweetened beverage intake and hyperuricemia risk.

**Supplementary Figure S25 –** Egger’s test of sugar-sweetened beverage intake and hyperuricemia risk.

**Supplementary Figure S26 –** Sensitivity analysis of sugar-sweetened beverage intake and hyperuricemia risk.

**Supplementary Figure S27 –** Funnel chart of tea intake and hyperuricemia risk.

**Supplementary Figure S28 –** Egger’s test of tea intake and hyperuricemia risk.

**Supplementary Figure S29 –** Sensitivity analysis of tea intake and hyperuricemia risk.

**Supplementary Table S9 –** Meta-regression results for tea consumption and hyperuricemia risk.

**Supplementary Table S10 –** Dose-response association of daily coffee intake (mL/day) and gout risk.

**Supplementary Table S11 –** Dose-response association of daily coffee intake (mL/day) and hyperuricemia risk.

**Supplementary Figure S30 –** Dose-response association of daily coffee intake (mL/day) and hyperuricemia risk in male.

**Supplementary Table S12 –** Dose-response association of daily coffee intake (mL/day) and hyperuricemia risk in male.

**Supplementary Figure S31 –** Dose-response association of daily coffee intake (mL/day) and hyperuricemia risk in female.

**Supplementary Table S13 –** Dose-response association of daily coffee intake (mL/day) and hyperuricemia risk in female.

**Supplementary Table S14 –** Dose-response association of alcohol intake (g/day) and gout risk.

**Supplementary Table S15 –** Dose-response association of daily alcohol intake (g/day) and hyperuricemia risk.

**Supplementary Figure S32 –** Dose-response association of daily alcohol intake (g/day) and hyperuricemia risk in male.

**Supplementary Table S16 –** Dose-response association of daily alcohol intake (g/day) and hyperuricemia risk in male.

**Supplementary Table S17 –** Dose-response association of daily sugar-sweetened beverage intake (mL/day) and gout risk.

**Supplementary Figure S33 –** Dose-response association of daily sugar-sweetened beverage intake (mL/day) and gout risk in male.

**Supplementary Table S18 –** Dose-response association of daily sugar-sweetened beverage intake (mL/day) and gout risk in male.

**Supplementary Figure S34 –** Dose-response association of daily sugar-sweetened beverage intake (mL/day) and gout risk in female.

**Supplementary Table S19 –** Dose-response association of daily sugar-sweetened beverage intake (mL/day) and gout risk in female.

**Supplementary Table S20 –** Dose-response association of daily sugar-sweetened beverage intake (mL/day) and hyperuricemia risk.

**Supplementary Table S21 –** Dose-response association of tea intake (mL/day) and gout risk.

**Supplementary Table S22 –** Dose-response association of tea intake (occasions/week) and hyperuricemia risk.

**Supplementary Methods 1 –** Database search strategy.

| database | Search Expression | |
| --- | --- | --- |
| PubMed | ("Hyperuricemia"[Title/Abstract] OR "Gouts"[Title/Abstract] OR "gout"[Title/Abstract] OR "Acid, Uric"[Title/Abstract] OR "2,6,8-Trihydroxypurine"[Title/Abstract] OR "Trioxopurine"[Title/Abstract] OR "Ammonium Acid Urate"[Title/Abstract] OR "Urate, Ammonium Acid"[Title/Abstract] OR "Potassium Urate"[Title/Abstract] OR "Urate, Potassium"[Title/Abstract] OR "Sodium Urate Monohydrate"[Title/Abstract] OR "Urate Monohydrate, Sodium"[Title/Abstract] OR "Monosodium Urate"[Title/Abstract] OR "Monosodium Urate Monohydrate"[Title/Abstract] OR "Urate Monohydrate, Monosodium"[Title/Abstract] OR "Sodium Acid Urate"[Title/Abstract] OR "Acid Urate, Sodium"[Title/Abstract] OR "Urate, Sodium Acid"[Title/Abstract] OR "Sodium Acid Urate Monohydrate"[Title/Abstract] OR "Sodium Urate"[Title/Abstract] OR "Urate, Sodium"[Title/Abstract] OR "Urate"[Title/Abstract] OR "Hyperuricemia"[MeSH Terms]) AND ("beverage"[Title/Abstract] OR "tea"[Title/Abstract] OR "alcohol"[Title/Abstract] OR "wine"[Title/Abstract] OR "beer"[Title/Abstract] OR "liquor"[Title/Abstract] OR "coffee"[Title/Abstract] OR "soda"[Title/Abstract] OR "soft drinks"[Title/Abstract] OR "diet"[Title/Abstract] OR "carbonated beverage"[Title/Abstract] OR "soda pop"[Title/Abstract] OR "cola beverage"[Title/Abstract] OR "cola drink"[Title/Abstract] OR "cordial"[Title/Abstract] OR "cordial beverage"[Title/Abstract] OR "cordial drink"[Title/Abstract] OR "flavoured water"[Title/Abstract] OR "artificial juices"[Title/Abstract]) | |
| Embase | ('hyperuricemia':ti,ab OR 'gouts':ti,ab OR 'gout':ti,ab OR 'uric acid':ti,ab OR '2,6,8-trihydroxypurine':ti,ab OR 'trioxopurine':ti,ab OR 'ammonium acid urate':ti,ab OR 'potassium urate':ti,ab OR 'sodium urate monohydrate':ti,ab OR 'monosodium urate':ti,ab OR 'monosodium urate monohydrate':ti,ab OR 'sodium acid urate':ti,ab OR 'sodium acid urate monohydrate':ti,ab OR 'sodium urate':ti,ab OR 'urate':ti,ab OR 'hyperuricemia'/exp) AND ('beverage':ti,ab OR 'tea':ti,ab OR 'alcohol':ti,ab OR 'wine':ti,ab OR 'beer':ti,ab OR 'liquor':ti,ab OR 'coffee':ti,ab OR 'soda':ti,ab OR 'soft drinks':ti,ab OR 'diet':ti,ab OR 'carbonated beverage':ti,ab OR 'soda pop':ti,ab OR 'cola beverage':ti,ab OR 'cola drink':ti,ab OR 'cordial':ti,ab OR 'cordial beverage':ti,ab OR 'cordial drink':ti,ab OR 'flavoured water':ti,ab OR 'artificial juices':ti,ab) | |
| Cochrane Library | #1= | (Hyperuricemia):ti,ab,kw OR (Gouts):ti,ab,kw OR (gout):ti,ab,kw OR (Acid, Uric):ti,ab,kw OR (Trioxopurine):ti,ab,kw OR (Ammonium Acid Urate):ti,ab,kw OR (Urate, Ammonium Acid):ti,ab,kw OR (Potassium Urate):ti,ab,kw OR (Urate, Potassium):ti,ab,kw OR (Sodium Urate Monohydrate):ti,ab,kw OR (Urate Monohydrate, Sodium):ti,ab,kw OR (Monosodium Urate):ti,ab,kw OR (Monosodium Urate Monohydrate):ti,ab,kw OR (Urate Monohydrate, Monosodium):ti,ab,kw OR (Sodium Acid Urate):ti,ab,kw OR (Acid Urate, Sodium):ti,ab,kw OR (Urate, Sodium Acid):ti,ab,kw OR (Sodium Acid Urate Monohydrate):ti,ab,kw OR (Sodium Urate):ti,ab,kw OR (Urate, Sodium):ti,ab,kw OR (Urate):ti,ab,kw |
|  | #2= | (beverage):ti,ab,kw OR (tea):ti,ab,kw OR (alcohol):ti,ab,kw OR (wine):ti,ab,kw OR (beer):ti,ab,kw OR (liquor):ti,ab,kw OR (coffee):ti,ab,kw OR (soda):ti,ab,kw OR (soft drinks):ti,ab,kw OR (diet):ti,ab,kw OR (carbonated beverage):ti,ab,kw OR (soda pop):ti,ab,kw OR (cola beverage):ti,ab,kw OR (cola drink):ti,ab,kw OR (cordial):ti,ab,kw OR (cordial beverage):ti,ab,kw OR (cordial drink):ti,ab,kw OR (flavoured water):ti,ab,kw OR (artificial juices):ti,ab,kw |
|  | #3= | #1 AND #2 |
| Web of Science | TS=(hyperuricemia OR gouts OR gout OR "uric acid" OR "2,6,8-trihydroxypurine" OR trioxopurine OR "ammonium acid urate" OR "potassium urate" OR "sodium urate monohydrate" OR "monosodium urate" OR "monosodium urate monohydrate" OR "sodium acid urate" OR "sodium acid urate monohydrate" OR "sodium urate" OR urate) AND ("beverage" OR "tea" OR "alcohol" OR "wine" OR "beer" OR "liquor" OR "coffee" OR "soda" OR "soft drinks" OR "diet" OR "carbonated beverage" OR "soda pop" OR "cola beverage" OR "cola drink" OR "cordial" OR "cordial beverage" OR "cordial drink" OR "flavoured water" OR "artificial juices") | |

**Supplementary Table S1 –** Explanation of Technical Terms and Abbreviations.

**Part 1.** Statistical terms

| **Term** | **Explanation** |
| --- | --- |
| Odds Ratio (OR) | The odds ratio (OR) is a fundamental metric employed in epidemiological research to assess the strength of the association between exposure factors and outcome events. It is defined as the ratio of the odds of the outcome occurring in the exposed group to the odds of the outcome occurring in the non-exposed group. Numerically, an OR of 1 signifies no association between exposure and outcome, an OR greater than 1 indicates an increased risk of the outcome due to exposure, and an OR less than 1 suggests a reduced risk of the outcome due to exposure. The reliability of the OR should be evaluated in the context of a 95% confidence interval. |
| 95% Confidence Interval (CI) | In the context of a meta-analysis examining influencing factors, the 95% confidence interval (CI) serves as a fundamental metric for assessing the reliability of research findings and the actual range of variation in effect size. Its pivotal role encompasses three primary aspects: First, it indicates the precision of the effect size estimation (e.g., odds ratio [OR], relative risk [RR]). A narrower interval suggests higher consistency and accuracy among the included studies, whereas a wider interval implies greater heterogeneity between studies or an insufficient sample size, thereby reducing the stability of the results. Second, the 95% CI is instrumental in determining the statistical significance of the effect. If the interval does not encompass the "null values" of the effect size (OR/RR=1, mean difference=0), it signifies that the relationship between the influencing factor and the outcome is statistically significant at the 95% confidence level. Third, the 95% CI aids in interpreting the practical significance of the findings. The interval range facilitates an intuitive understanding of the true potential range of the effect size, thereby mitigating the risk of biased judgments that may arise from relying exclusively on a singular effect size value. This approach offers more comprehensive evidential support for subsequent decision-making processes. |
| Heterogeneity | In the context of meta-analysis, heterogeneity pertains to the variability among included studies, which can manifest across various dimensions such as study outcomes, methodologies, population characteristics, or intervention strategies. This variability fundamentally indicates that the effect size of each individual study cannot be adequately accounted for by a single common effect. The concept of heterogeneity encompasses statistical, clinical, and methodological dimensions. Statistical heterogeneity is assessed by quantifying the degree of variation in effect sizes, with the I² statistic frequently employed as a measure; for instance, an I² value exceeding 50% is indicative of moderate to high heterogeneity. |
| Nonlinear Dose-Response Relationship | In research, a nonlinear dose-response relationship describes the association between exposure dose and outcome events, characterized by complex curve shapes or non-uniform variations rather than a straightforward proportional increase or decrease in outcome with increasing dose. This relationship may manifest as rapid enhancement followed by a plateau, an initial increase followed by a decrease, or abrupt changes in effect at specific dose thresholds. Such complexity indicates that the association between exposure dose and outcome is influenced by changes in slope, saturation points, thresholds, or other intricate mechanisms inherent in the dose-response curve. Consequently, simple linear models are inadequate for capturing this relationship, necessitating the use of nonlinear regression and other advanced methods to accurately elucidate the true impact pattern of exposure dose on outcomes. |

**Part 2. Abbreviations**

| **Abbreviation** | **Full term** |
| --- | --- |
| PRISMA | Preferred Reporting Items for Systematic Reviews and Meta-Analyses |
| PECOTS | Population, Exposure, Comparator, Outcome, Time frame, and Study design |
| HUA | Hyperuricemia |
| SUA | Serum uric acid |
| SSBs | Sugar-sweetened beverages |
| OR | Odds ratio |
| RR | Risk ratio |
| HR | Hazard ratio |
| CI | Confidence interval |
| BMI | Body mass index |
| FFQ | Food frequency questionnaire |
| NOS | Newcastle-Ottawa Scale |
| AHRQ | Agency for Healthcare Research and Quality |
| ICD | International Classification of Diseases |
| ACR | American College of Rheumatology |
| eGFR | Estimated glomerular filtration rate |
| TDI | Townsend Deprivation Index |
| T2DM | Type 2 diabetes mellitus |
| ATP | Adenosine triphosphate |
| URAT1 | Urate transporter 1 |
| NLRP3 | NLR family pyrin domain containing 3 |

**Supplementary Table S2** – Population, Exposure, Comparator, Outcome, Time frame, Study design (PECOTS) approach

| **Participants (P)** | Generally population from any racial or ethnic background. |
| --- | --- |
| **Exposure (E)** | Explicit assessment of the intake of different beverages (including alcohol, coffee, tea, sugar-sweetened beverages, etc.). |
| **Comparator (C)** | Non-consumers or the lowest category of intake for the specific beverage. |
| **Outcomes (O)** | Confirmed diagnosis of hyperuricemia or gout. Studies must provide relevant effect sizes (relative risk [RR], hazard ratio [HR], odds ratio [OR]) and their 95% confidence intervals (CIs) |
| **Time Frame (T)** | For prospective cohort studies, a minimum follow-up duration of 2 years. No duration restriction for cross-sectional and case-control studies. |
| **Study design (S)** | Observational studies, including cross-sectional, cohort, and case-control studies |

**Supplementary Table S3** – Exposure unit conversion for dose-response analysis

| **Study** | **Original unit** | **Definition of unit** | **Conversion formula** | **Analytical unit** | **Citable source** |
| --- | --- | --- | --- | --- | --- |
| **COFFEE — Gout (unified scale: mL/day)** | | | | | |
| Choi 2007 | cups/day | 1 cup = 237 mL = 8 fl oz cup | mL/day = cups/day × 237 | mL/day | Original study |
| Choi 2010 | mL/day and cups/day | 1 cup = 237 mL = 8 fl oz cup | mL/day = cups/day × 237 | mL/day | Original study |
| **COFFEE — Hyperuricaemia (unified scale: mL/day)** | | | | | |
| Pham NM 2010 | cups/day | 1 cup = 150 mL | mL/day = cups/day × 150 | mL/day | Original paper |
| **ALCOHOL — Gout (unified scale: g ethanol/day)** | | | | | |
| Choi 2004 | g (ethanol)/day | None | None | g (ethanol)/day | Original paper |
| Cea Soriano L 2011 | units/week | 1 unit = 10 mL of pure ethanol = 8 grams of alcohol | g (ethanol)/day = units/week × 8 ÷ 7 | g (ethanol)/day | Original paper |
| Kuo CF 2016 | units/week | 1 unit = 10 mL of pure ethanol = 8 grams of alcohol | g (ethanol)/day = units/week × 8 ÷ 7 | g (ethanol)/day | Original paper |
| **ALCOHOL — Hyperuricaemia (unified scale: g ethanol/day)** | | | | | |
| Sugie T 2005 | g/day | None | None | g ethanol/day | Original paper |
| Ryu S 2011 | g/day | None | None | g ethanol/day | Original paper |
| Li Z 2016 | drinks/day | 1 drink = 15 g pure alcohol | g/day = drinks/day × 15 | g ethanol/day | Original paper |
| **SSB — Gout (unified scale: mL/day)** | | | | | |
| Choi HK 2008 | servings/day | 1 serving = 355 mL = 12 fl oz | mL/day = servings/day × 355 | mL/day | Original paper |
| Choi HK 2010 | servings/day | 1 serving = 355 mL = 12 fl oz | mL/day = servings/day × 355 | mL/day | Original paper |
| **SSB — Hyperuricaemia (unified scale: mL/day)** | | | | | |
| Meneses-León J 2020 | servings/week | 1 serving = 355 mL | mL/day = servings/week × 355 ÷ 7 | mL/day | Original paper |
| Meneses-León J 2014 | servings/day | 1 serving = 355 mL | mL/day = servings/day × 355 | mL/day | Original paper |
| **TEA — Gout (unified scale: mL/day)** | | | | | |
| Choi HK 2007 | cups/day | 1 cup = 237 mL | mL/day = cups/day × 237 | mL/day | Original paper |
| Choi HK 2010 | cups/day | 1 cup = 237 mL | mL/day = cups/day × 237 | mL/day | Original paper |
| **TEA — Hyperuricaemia (unified scale:occasions/week)** | | | | | |
| Li X 2015 | times/week | None | None | occasions/week | Original paper |
| Lee JS 2021 | Consumption occasions per day, week or month | 1 week = 7 days  1 month = 30 days | occasions/week = times/day × 7  occasions/week = monthly occasions × 7 ÷ 30 | occasions/week | Original paper |
| Li R 2022 | Frequency categories with monthly and weekly boundaries | 1 week = 7 days  1 month = 30 days | occasions/week = times/day × 7  occasions/week = monthly occasions × 7 ÷ 30 | occasions/week | Original paper |

**Supplementary Table S4 –** Demographic characteristics of included studies.

| **Author** | **Year** | **Country/Region** | **N** | **Study design** | **Age** | **Beverage Type** | **Evaluation Methods for Exposure** | **Outcome** | **Outcome Definition** | **Adjustment Variables** |
| --- | --- | --- | --- | --- | --- | --- | --- | --- | --- | --- |
| Choi HK | 2007 | USA | M: 45869 | Cohort study | 54±10 | Coffee, Tea | FFQ | Gout | ACR criteria | Age, total energy intake, BMI, diuretic use, history of hypertension, history of renal failure, alcohol, total meat |
| Choi HK | 2010 | USA | F: 89433 | Cohort study | 46±7 | Coffee, Tea | FFQ | Gout | ACR criteria | Age, total energy intake, BMI, menopause, use of hormonal replacement, diuretic use, history of hypertension, and intakes of alcohol, sugar-sweetened soft drinks, total meats, seafood, chocolate, dairy foods, total vitamin C, and beverages |
| Guo H | 2023 | UK | M: 203003 F: 244655 | Cohort study | 56.20±8.13 | Coffee, Tea | Questionnaire | Gout | ICD-10 | Age, sex, income, TDI, ethnicity, BMI, qualification, smoking status, alcohol status, total physical activity level, sedentary time, duration of sleep, water intake, intake of processed meat, fresh fruits and vegetables, fish |
| Hutton J | 2018 | Europe | 130966 | Cross-sectional study | 56.7±7.98 | Coffee | FFQ | Gout | NR | Age, sex, body mass index, hypertension, kidney disease, diabetes, meat intake, fish intake, cheese intake, tea intake, fruit intake, vegetable intake, bread intake, cereal intake, beer/spirits |
| Bae J | 2015 | Korea | M: 3564 F: 5836 | Cross-sectional study | M: 62.5±9.6  F: 61.6±9.8 | Coffee, Tea | FFQ | Hyperuricemia | SUA>7.0 mg/dL(M), SUA>6.0 mg/dL(F) | Age, education, marital status, cigarette smoking, alcohol drinking, regular exercise, body mass index, triglyceride, fasting serum glucose, hypertension medication, and glomerular filtration rate, total energy, vitamin C, meat intake, seafood intake, dairy food intake, soft drink intake, added sugar in coffee, and added cream in coffee |
| Pham NM | 2010 | Japan | M: 4964 F: 6698 | Cross-sectional study | M: 62.6±6.8  F: 62.1±6.7 | Coffee | FFQ | Hyperuricemia | SUA>7.0 mg/dL(M), SUA>6.0 mg/dL(F) | Age, BMI, smoking, alcohol use, work-related physical activity and leisure-time physical activity, hypertension, diabetes, eGFR, and seafood intake |
| Teng GG | 2013 | Singapore | M:214 F:269 | Cohort study | 57.6±7.9 | Coffee, Green tea | FFQ | Hyperuricemia | SUA>6 mg/dL | Cholesterol, creatinine, glycosylated hemoglobin, triglycerides, age, sex, BMI, education, cigarette smoking status, physical activity status, hypertension, dairy products, red meat, fish, and other beverages |
| Agoons DD | 2021 | USA | F: 2139 | Cross-sectional study | 31.2±9.6 | Coffee | FFQ | Hyperuricemia | SUA>5.7 mg/dL | Age, alcohol consumption, smoking, diabetes, hypertension, eGFR, BMI |
| Lee JS | 2021 | Korea | M: 57596 F: 110156 | Cross-sectional study | 53.1±8.3 | Coffee, SSB, Green tea | FFQ | Hyperuricemia | SUA>7.0 mg/dL(M), SUA>6.0 mg/dL(F) | Age, sex, BMI, income, past medical histories, smoking,alcohol consumption, nutritional intake, frequency of coffee, green tea, and soft drinks |
| Li QH | 2022 | China | M: 620 | Cross-sectional study | 42.7±14.2 | Coffee, Alcohol, SSB, Tea | FFQ | Hyperuricemia | SUA ≥600 µmol/L | Age, duration of gout, family history, BMI, eGFR, tophi, comorbidities (i.e., hypertension, T2DM, dyslipidemia, and coronary heart disease, current medication) (i.e., aspirin, diuretics, and lipid-lowering medication) |
| Im PK | 2023 | China | 512724 | Cohort study | 52±10.7 | Alcohol | NR | Gout | ICD-10 | Age at risk, study area, education, smoking |
| Lyu JQ | 2024 | UK | M: 217964 F: 271257 | Cohort study | M: 56.0 ± 8.2  F: 56.0 ± 8.0 | Alcohol | Questionnaire | Gout | ICD-9 and ICD-10 | Age, racial and ethnic group, Townsend Deprivation Index, smoking, physical activity, hypertension, dyslipidemia, diabetes, eGFR, diuretic use, major food groups, BMI |
| Choi HK | 2004 | USA | M: 47150 | Cohort study | 54 ± 10 | Alcohol | FFQ | Gout | ACR criteria | Age, total energy intake, BMI, diuretic use, history of hypertension, history of renal failure, intake of total meats, seafood, purine-rich vegetables, dairy foods, fluid |
| Lyu L | 2003 | China | 184 | Case-control study | 20-70 | Alcohol | FFQ | Gout | ACR criteria | Age, educational level |
| Cohen SD | 2008 | USA | 259209 | Cohort study | 65.36 ± 15.6 | Alcohol | NR | Gout | NR | Black race, older age, female gender, hypertension, ischemic heart disease, congestive heart failure, alcohol use, a history of diabetes, smoking, and peripheral vascular disease |
| Williams PT | 2008 | USA | M: 28990 | Cohort study | 44.7 ± 10.9 | Alcohol | Questionnaire | Gout | NR | Meat, fish, and fruit, age, hypertension, and BMI |
| Bhole V | 2010 | USA | M: 1951 F: 2476 | Cohort study | M: 46 F: 47 | Alcohol | Self-report | Gout | NR | Age, Education level, Body mass index, Alcohol consumption, Hypertension, Use of diuretics, Blood glucose level, Blood cholesterol level, and Menopausal status. |
| Cea Soriano L | 2011 | UK | 74768 | Case-control study | 20-89 | Alcohol | Records | Gout | NR | Sex, age, calendar year, GP visits, BMI, alcohol consumption, IHD, hypertension, hyperlipidemia, diabetes, chronic renal failure, diuretics |
| Lee CH | 2013 | Korea | 36246 | Case-control study | 55.5±10.2 | Alcohol | Questionnaire | Gout | ICD-10 | Age, sex, BMI, blood pressure, blood sugar, total cholesterol, urine protein, uric acid |
| Rasheed H | 2013 | Europe | 2636 | Case-control study | NR | Alcohol | FFQ | Gout | ACR criteria | Sex, age, BMI, sugar-sweetened beverage consumption |
| Wang Y | 2013 | China | 659 | Prospective cohort study | 47±22 | Alcohol | 7-day dietary record | Gout | ACR criteria | Gender, education, category of drinks, labor intensity, family history of gout, diabetes, hypertension, smoking, obesity, age |
| Kuo CF | 2016 | Britain | 78222 | Case-control study | 62.2±15.1 | Alcohol | Medical records | Gout | ACR criteria | Age, gender, index year |
| Zhang M | 2022 | China | 355212 | Cross-sectional study | 43.7 ± 16.1 | Alcohol | Questionnaire | Hyperuricemia | SUA>420 μmol/L | NR |
| Sugie T | 2005 | Japan | M: 715 | Cross-sectional study | 35-60 | Alcohol | Questionnaire | Hyperuricemia | SUA>7.0 mg/dL(M) | Age, BMI, intake of snacks, smoking habits |
| Ryu S | 2011 | Korea | M: 10802 | Cohort study | 37.3±5.0 | Alcohol | Questionnaire | Hyperuricemia | SUA>7.0 mg/dL(M) | Age, body mass index, blood pressure, fasting glucose, hsCRP, eGFR, triglycerides, total cholesterol, HDL, GGT, fatty liver |
| Nakamura K | 2012 | Japan | M: 3310 | Cohort study | 38.5±9.6 | Alcohol | Questionnaire | Hyperuricemia | SUA>7.1 mg/dL(M) | Age, baseline serum uric acid, body mass index, smoking habits, exercise habits, serum creatinine, systolic blood pressure, medication for hypertension, serum total cholesterol, medication for hypercholesterolemia, HbA1c, medication for diabetes |
| Xiong Z | 2013 | China | F: 856 | Cross-sectional study | 68.96±6.9 | Alcohol | FFQ | Hyperuricemia | SUA>416 μmol/L | Age, kcal/day, WHR, BMI, physical activity, meat and fish intake, hypertension |
| Makinouchi T | 2016 | Japan | M: 8097 | Prospective cohort study | 42.9±12.0 | Alcohol | Questionnaire | Hyperuricemia | SUA>7.0 mg/dL(M) | Age, body mass index, mean arterial pressure, triglycerides, HbA1c, AST, creatinine, job schedule type, tobacco consumption, habitual exercise |
| Li Z | 2016 | China | M: 4997 F: 6042 | Cross-sectional study | M: 54.4±10.8 F: 53.4±10.3 | Alcohol | Questionnaire | Hyperuricemia | SUA≥416 μmol/L(M), SUA ≥357 μmol/L(F) | Age, race, education, income, diet scores, smoking status, activity level, obesity, hypertension, dyslipidemia, diabetes, medication used, menopausal status |
| Zhong L | 2022 | China | M: 5065 F: 6110 | Cross-sectional study | 35-74 | Alcohol, Tea | Questionnaire | Hyperuricemia | SUA>420 μmol/L | Education level, history of CHD, hypertension, smoking, BMI, central obesity, hyper-TG, hyper-TC, hypo-HDL-C, abnormal AST, diabetes |
| Ding X | 2023 | China | M: 10512 F: 11937 | Cross-sectional study | 51.5±11.8 | Alcohol, Tea | Questionnaire | Hyperuricemia | SUA>7.0 mg/dL(M), SUA>6.0 mg/dL(F) | Sex, age, socioeconomic status, family history of diabetes, smoking, drinking beverage, physical activity, insufficient vegetable and fruit intake, spicy food, hemp diet |
| Wu Y | 2023 | China | M: 3418 F: 3665 | Cross-sectional study | ≥18 | Alcohol | Questionnaire | Hyperuricemia | SUA>7.0 mg/dL(M), SUA>6.0 mg/dL(F) | Age, area, smoking, BMI, waist circumference, education level, physical activity, creatinine level, CRP, blood pressure, dietary intakes |
| Ji-Hong Shao | 2004 | China | 1144 | Case-control study | 54.2±12.4 | Alcohol | Questionnaire | Hyperuricemia | SUA>7.0 mg/dL(M), SUA>6.0 mg/dL(F) | NR |
| Silva MTD | 2020 | Brazil | 14320 | Cross-sectional study | 52±9 | Alcohol | FFQ | Hyperuricemia | SUA≥6.8 mg/dL | Age, body mass index, glomerular filtration rate, use of medications (losartan, diuretics, acetylsalicylic acid, beta-blockers, and estrogen for women), other groups of foods and alcoholic beverages. |
| So MW | 2020 | Korea | M: 4300 F: 5875 | Cross-sectional study | 47 | Alcohol | Single 24-hour recall method | Hyperuricemia | SUA ≥6.0 mg/dL | Age, sex, obesity, hypertension, chronic kidney disease, elevated high sensitivity C-reactive protein level, intake of energy and water |
| Choi HK | 2008 | USA | M: 46393 | Prospective cohort study | 54.3 | SSB | FFQ | Gout | ACR criteria | Age, total energy intake, alcohol intake, body mass index, use of diuretics, history of hypertension, history of chronic renal failure, meats, seafood, purine rich vegetables, dairy foods, total vitamin C |
| Choi HK | 2010 | USA | F: 78906 | Prospective cohort study | 50.5±7.1 | SSB | FFQ | Gout | ACR criteria | Age, total energy intake, alcohol, body mass index, menopause status, use of hormone therapy, use of diuretics, history of hypertension, coffee intake, meats, seafood, dairy foods, total vitamin C |
| Dalbeth N | 2015 | New Zealand & USA | 12870 | Cross-sectional study | 49.5±10.1 | SSB | FFQ | Gout | ACR criteria | ethnicity, age, sex, fruit intake, triglycerides, kidney disease, and hypertension |
| Rai SK | 2024 | USA | M: 43703 F: 78976 | Prospective cohort study | M: 53.8±9.8 F: 50.9±7.2 | SSB | FFQ | Gout | ACR criteria | Total energy intake, BMI, history of hypertension, diuretic use, physical activity, alcohol intake (women additionally adjusted for oral contraceptive use, menopausal status, and hormone therapy use) |
| Zhang T | 2020 | China | M: 13013 F: 12494 | Cross-sectional study | M: 42.0  F: 39.5 | SSB | FFQ | Hyperuricemia | SUA ≥7.0 mg/dL (M), ≥6.0 mg/dL (F) | Age, BMI, smoking and drinking status, physical activity, educational level, employment status, household incomes, MetS, total energy intake, meat, seafood, carbohydrate, total fat, total vegetables, total fruits, sweet foods, EPA, DHA, other beverages (green tea, black tea, coffee, oolong tea, juice) |
| Siqueira JH | 2018 | Brazil | M: 3325 F: 3848 | Cross-sectional study | 50±8.4 | SSB | FFQ | Hyperuricemia | SUA >7.0 mg/dL (M), >6.0 mg/dL (F) | Age, education, per capita income, smoking, physical activity, alcohol consumption, GFR, waist circumference, total energy, meat intake, seafood intake, coffee intake, dairy food intake, daily fruits, daily vegetables, use of vitamin supplements, Vitamin C, other beverages (women additionally adjusted for menopause and hormone therapy) |
| Siqueira JH | 2021 | Brazil | M: 4151 F: 5921 | Cohort study | 51.3±8.9 | SSB | FFQ | Hyperuricemia | SUA ≥7.0 mg/dL (M), ≥5.7 mg/dL (F) | Age, education, smoking, physical activity, alcohol use, consumption of fruits and vegetables, meats and seafood, dairy products, coffee, dietary vitamin C, energy, GFR, presence of hypertension and diabetes, use of diuretics, BMI delta (women additionally adjusted for menopause and hormone replacement therapy) |
| Meneses-León J | 2020 | Mexico | M: 322  F: 978 | Prospective cohort study | 45.8±12.9 | SSB | FFQ | Hyperuricemia | SUA ≥7.0 mg/dL (M), ≥5.7 mg/dL (F) | Age, smoking status, physical activity, dietary intake of meat, seafood, alcohol, sex and BMI |
| Meneses-Leon J | 2014 | Mexico | M: 1956 F: 4749 | Cross-sectional study | M: 42.6±11.6  F: 43.0±12.2 | SSB | FFQ | Hyperuricemia | SUA ≥7.0 mg/dL (M), ≥5.8 mg/dL (F) | Age, tobacco use, physical activity, weight change, calorie intake, alcohol consumption, total carbohydrates, total meats, seafood, total fruits, dairy products, coffee, vitamin C, BMI (women additionally adjusted for menopause and postmenopausal hormone use) |
| Lin WT | 2012 | China | 2727 | Cross-sectional study | 13.7 | SSB | FFQ | Hyperuricemia | SUA >7 mg/dL (M), >6 mg/dL (F) | Study area, age, gender, physical activity, total calories, intake of meat, seafood, fruit, fried food, food with jelly/honey, alcohol drinking, cigarette smoking, BMI |
| Lee SM | 2024 | Korea | M: 1066 F: 1815 | Cross-sectional study | M: 40.6±0.5  F: 41.5±0.4 | SSB | FFQ | Hyperuricemia | SUA ≥6.7 mg/dL (M), ≥4.8 mg/dL (F) | Age, personal income, educational level, physical activity, hypertension status, diabetes status, hypercholesterolemia status, smoking history, alcohol consumption history, SBP, DBP, waist circumference, BMI, fasting blood glucose, triglyceride, HDL-C, daily intake of energy, protein, saturated fat, carbohydrates |
| Bomback AS | 2010 | USA | 15745 | Prospective cohort study | NR | SSB | FFQ | Hyperuricemia | SUA >7.0 mg/dL (M), >5.7 mg/dL (F) | Age, sex, animal protein intake, caffeine intake, hypertension, body mass index, renal function, current tobacco and alcohol use, ARIC field center, and race |
| Bae J | 2013 | Korea | M: 3564 F: 5836 | Cross-sectional study | M: 62.5±9.6  F: 61.6±9.8 | SSB | FFQ | Hyperuricemia | SUA >7.0 mg/dL (M), >6.0 mg/dL (F) | Age, education, marital status, cigarette smoking, alcohol drinking, regular exercise, BMI, anti-hypertensive medication, GFR, total energy, vitamin C, meat, seafood, dairy, and coffee intake |
| Batt C | 2014 | New Zealand | 8709 | Cross-sectional study | 52.2 | SSB | Questionnaire | Gout | ACR criteria | Age, sex, BMI, alcohol consumption, fruit consumption, kidney disease, and hypertension |
| Li X | 2015 | China | M: 720 F: 652 | Cross-sectional study | 37.7±9.7 | Tea | FFQ | Hyperuricemia | SUA >416 µmol/L (M), >357 µmol/L (F) | Age, smoking, drinking status |
| Li R | 2022 | China | M: 4440 F: 3204 | Cross-sectional study | 34.7±8.9 | Tea | Questionnaire | Hyperuricemia | SUA >420 µmol/L (M), >360 µmol/L (F) | Age, BMI, SCr, CHOL, TG, LDL-C, HDL-C, FBG, hypertension, educational level, smoking status, alcohol consumption, salty diet, midnight snacking, overeating, frequency of dining out, barbecue/fried food intake, corned/smoked food intake |
| Zeng J | 2020 | China | 1488 | Cross-sectional study | 37.7±9.6 | Tea | FFQ | Hyperuricemia | SUA >420 µmol/L (M), >360 µmol/L (F) |  |

NR: Not Reported

**Supplementary Table S5 –** Agency for Healthcare Research and Quality (AHRQ) assessment of the quality of cross-sectional studies

| **Cross-sectional studies** | **Item 1** | **Item 2** | **Item 3** | **Item 4** | **Item 5** | **Item 6** | **Item 7** | **Item 8** | **Item 9** | **Item 10** | **Item 11** | **Total Score** | **Quality Level** |
| --- | --- | --- | --- | --- | --- | --- | --- | --- | --- | --- | --- | --- | --- |
| Bae J (2015) | 1 | 1 | 1 | 1 | 0 | 1 | 1 | 1 | 1 | 1 | 0 | 9 | High |
| Pham NM (2010) | 1 | 1 | 1 | 1 | 0 | 1 | 1 | 1 | 1 | 1 | 0 | 9 | High |
| Hutton J (2018) | 1 | 1 | 1 | 1 | 0 | 1 | 1 | 1 | 1 | 1 | 0 | 9 | High |
| Agoons DD (2021) | 1 | 1 | 1 | 1 | 0 | 1 | 1 | 1 | 1 | 1 | 0 | 9 | High |
| Lee JS (2021) | 1 | 1 | 1 | 1 | 0 | 1 | 1 | 1 | 1 | 1 | 0 | 9 | High |
| Li QH (2022) | 1 | 1 | 1 | 1 | 0 | 1 | 1 | 1 | 1 | 1 | 0 | 9 | High |
| Zhang M (2022) | 1 | 1 | 1 | 1 | 0 | 1 | 1 | 1 | 1 | 1 | 0 | 9 | High |
| Sugie T (2005) | 1 | 1 | 1 | 1 | 0 | 1 | 1 | 1 | 1 | 1 | 0 | 9 | High |
| Xiong Z (2013) | 1 | 1 | 1 | 1 | 0 | 1 | 1 | 1 | 1 | 1 | 0 | 9 | High |
| Li Z (2016) | 1 | 1 | 1 | 1 | 0 | 1 | 1 | 1 | 1 | 1 | 0 | 9 | High |
| Zhong L (2022) | 1 | 1 | 1 | 1 | 0 | 1 | 1 | 1 | 1 | 1 | 0 | 9 | High |
| Ding X (2023) | 1 | 1 | 1 | 1 | 0 | 1 | 1 | 1 | 1 | 1 | 0 | 9 | High |
| Wu Y (2023) | 1 | 1 | 1 | 1 | 0 | 1 | 1 | 1 | 1 | 1 | 0 | 9 | High |
| Silva MTD (2020) | 1 | 1 | 1 | 1 | 0 | 1 | 1 | 1 | 1 | 1 | 0 | 9 | High |
| So MW (2020) | 1 | 1 | 1 | 1 | 0 | 1 | 1 | 1 | 1 | 1 | 0 | 9 | High |
| Zhang T (2020) | 1 | 1 | 1 | 1 | 0 | 1 | 1 | 1 | 1 | 1 | 0 | 9 | High |
| Siqueira JH (2018) | 1 | 1 | 1 | 1 | 0 | 1 | 1 | 1 | 1 | 1 | 0 | 9 | High |
| Meneses-Leon J (2014) | 1 | 1 | 1 | 1 | 0 | 1 | 1 | 1 | 1 | 1 | 0 | 9 | High |
| Lin WT (2012) | 1 | 1 | 1 | 1 | 0 | 1 | 1 | 1 | 1 | 1 | 0 | 9 | High |
| Lee SM (2024) | 1 | 1 | 1 | 1 | 0 | 1 | 1 | 1 | 1 | 1 | 0 | 9 | High |
| Bae J (2013) | 1 | 1 | 1 | 1 | 0 | 1 | 1 | 1 | 1 | 1 | 0 | 9 | High |
| Batt C (2014) | 1 | 1 | 1 | 1 | 0 | 1 | 1 | 1 | 1 | 1 | 0 | 9 | High |
| Dalbeth N (2015) | 1 | 1 | 1 | 1 | 0 | 1 | 1 | 1 | 1 | 1 | 0 | 9 | High |
| Li X (2015) | 1 | 1 | 1 | 1 | 0 | 1 | 1 | 1 | 1 | 1 | 0 | 9 | High |
| Li R (2022) | 1 | 1 | 1 | 1 | 0 | 1 | 1 | 1 | 1 | 1 | 0 | 9 | High |
| Zeng J (2020) | 1 | 1 | 1 | 1 | 0 | 1 | 1 | 1 | 1 | 1 | 0 | 9 | High |

**Supplementary Table S6 –** Newcastle-Ottawa Scale (NOS) Assessment of Cohort Study Quality

| **Cohort Studies** | **Selection** | | | | **Comparability** | **Outcome** | | | |
| --- | --- | --- | --- | --- | --- | --- | --- | --- | --- |
|  | **Exposed Cohort** | **Non-exposed Cohort** | **Exposure Ascertainment** | **Outcome of Interest** |  | **Outcome Assessment** | **Follow-up Duration** | **Adequacy of Follow-up** | **Total Score** |
| Choi HK (2007) | 1 | 1 | 1 | 1 | 2 | 1 | 1 | 1 | 9 |
| Choi HK (2010) | 1 | 1 | 1 | 1 | 2 | 1 | 1 | 1 | 9 |
| Guo H (2023) | 1 | 1 | 1 | 1 | 2 | 1 | 1 | 1 | 9 |
| Teng (2013) | 1 | 1 | 1 | 0 | 2 | 1 | 0 | 0 | 6 |
| Im PK (2023) | 1 | 1 | 1 | 1 | 2 | 1 | 1 | 1 | 9 |
| Lyu JQ (2024) | 1 | 1 | 1 | 1 | 2 | 1 | 1 | 1 | 9 |
| Choi HK (2004) | 1 | 1 | 1 | 1 | 2 | 1 | 1 | 1 | 9 |
| Cohen SD (2008) | 1 | 1 | 1 | 1 | 2 | 1 | 1 | 1 | 9 |
| Williams PT (2008) | 1 | 1 | 1 | 1 | 2 | 0 | 1 | 1 | 8 |
| Bhole V (2010) | 1 | 1 | 1 | 1 | 2 | 1 | 1 | 1 | 9 |
| Ryu S (2011) | 1 | 1 | 1 | 1 | 2 | 1 | 1 | 1 | 9 |
| Nakamura K (2012) | 1 | 1 | 1 | 1 | 2 | 1 | 1 | 1 | 9 |
| Wang Y (2013) | 1 | 1 | 1 | 1 | 2 | 1 | 1 | 1 | 9 |
| Makinouchi T (2016) | 1 | 1 | 1 | 1 | 2 | 1 | 1 | 1 | 9 |
| Choi HK (2008) | 1 | 1 | 1 | 1 | 2 | 1 | 1 | 1 | 9 |
| Choi HK (2010) | 1 | 1 | 1 | 1 | 2 | 1 | 1 | 1 | 9 |
| Siqueira JH (2021) | 1 | 1 | 1 | 1 | 2 | 1 | 1 | 1 | 9 |
| Meneses-Leon J (2020) | 1 | 1 | 1 | 0 | 2 | 1 | 1 | 0 | 7 |
| Bomback AS (2010) | 1 | 1 | 1 | 1 | 2 | 1 | 1 | 1 | 9 |
| Rai SK (2024) | 1 | 1 | 1 | 1 | 2 | 1 | 1 | 1 | 9 |

**Supplementary Table S7 –** Newcastle-Ottawa Scale (NOS) Assessment of Case-control studies

| **Case-control Studies** | **Selection** | | | | **Comparability** | **Exposure** | | | |
| --- | --- | --- | --- | --- | --- | --- | --- | --- | --- |
|  | **Case definition** | **Representativeness of cases** | **Selection of controls** | **Definition of controls** |  | **Assessment of Exposure** | **Same method for cases and controls** | **Non-response rate** | **Total Score** |
| Lyu L-C (2003) | 0 | 1 | 1 | 0 | 2 | 1 | 0 | 0 | 5 |
| Cea Soriano (2011) | 1 | 1 | 1 | 1 | 2 | 1 | 1 | 1 | 9 |
| Lee CH (2013) | 1 | 1 | 1 | 1 | 2 | 1 | 0 | 0 | 7 |
| Rasheed H (2013) | 0 | 1 | 1 | 0 | 2 | 1 | 0 | 0 | 5 |
| Kuo CF (2016) | 1 | 1 | 1 | 1 | 2 | 1 | 1 | 1 | 9 |
| Shao JH (2004) | 1 | 1 | 1 | 0 | 2 | 1 | 0 | 0 | 6 |

**Supplementary Figure S1 –** Funnel chart of coffee intake and gout risk

**Supplementary Figure S2 –** Egger’s test of coffee intake and gout risk

**Supplementary Figure S3 –** Sensitivity analysis of coffee intake and gout risk

**Supplementary Figure S4 –** Funnel chart of coffee intake and hyperuricemia risk

**Supplementary Figure S5 –** Egger’s test of coffee intake and hyperuricemia risk

**Supplementary Figure S6 –** Sensitivity analysis of coffee intake and hyperuricemia risk

**Supplementary Figure S7 –** Subgroup analysis examines the relationship between coffee intake and hyperuricemia risk.

**Supplementary Figure S8 –** Funnel chart of alcohol intake and gout risk.

**Supplementary Figure S9 –** Egger’s test of alcohol intake and gout risk.

**Supplementary Figure S10 –** Sensitivity analysis of alcohol intake and gout risk.

**Supplementary Figure S11 –** Alcohol categories subgroup analysis examines the relationship between alcohol intake and gout risk.

**Supplementary Table S8 –** Meta-regression results for alcohol consumption and gout risk.

| Moderator | Coding | β (95% CI) | p-value | R² (%) |
| --- | --- | --- | --- | --- |
| Geographic region | Asia = 1; Western = 0 | −0.427 (−1.158, 0.305) | 0.291 | 60.8 |
| Study design | Cohort = 1; Case-control/cross-sectional = 0 | −0.182 (−0.883, 0.519) | 0.626 | 38.9 |
| Sex composition | Male-only = 1; Mixed/female = 0 | −0.114 (−0.914, 0.686) | 0.789 | 34.4 |

**Supplementary Figure S12 –** Forest plot of alcohol intake and gout risk (HR).

**Supplementary Figure S13 –** Funnel chart of alcohol intake and gout (HR).

**Supplementary Figure S14 –** Egger’s test of alcohol intake and gout risk (HR).

**Supplementary Figure S15 –** Sensitivity analysis of alcohol intake and gout risk (HR).

**Supplementary Figure S16 –** Funnel chart of alcohol intake and hyperuricemia risk.

**Supplementary Figure S17 –** Egger’s test of alcohol intake and hyperuricemia risk.

**Supplementary Figure S18 –** Sensitivity analysis of alcohol intake and hyperuricemia risk.

**Supplementary Figure S19 –** Alcohol categories subgroup analysis in males examines the relationship between alcohol intake and hyperuricemia risk.

**Supplementary Figure S20 –** Alcohol categories subgroup analysis in females examines the relationship between alcohol intake and hyperuricemia risk.

**Supplementary Figure S21 –** Funnel chart of sugar-sweetened beverage intake and gout risk.

**Supplementary Figure S22 –** Egger’s test of sugar-sweetened beverage intake and gout risk.

**Supplementary Figure S23 –** Sensitivity analysis of sugar-sweetened beverage intake and gout risk.

**Supplementary Figure S24 –** Funnel chart of sugar-sweetened beverage intake and hyperuricemia risk.

**Supplementary Figure S25 –** Egger’s test of sugar-sweetened beverage intake and hyperuricemia risk.

**Supplementary Figure S26 –** Sensitivity analysis of sugar-sweetened beverage intake and hyperuricemia risk.

**Supplementary Figure S27 –** Funnel chart of tea intake and hyperuricemia risk.

**Supplementary Figure S28 –** Egger’s test of tea intake and hyperuricemia risk.

**Supplementary Figure S29 –** Sensitivity analysis of tea intake and hyperuricemia risk.

**Supplementary Table S9 –** Meta-regression results for tea consumption and hyperuricemia risk.

| Moderator | Coding | β (95% CI) | p-value | R² (%) |
| --- | --- | --- | --- | --- |
| Tea type | Green tea = 1; Mixed/unspecified = 0 | +0.081 (-0.393, 0.555) | 0.738 | 0.0 |
| Country | China = 1; Other = 0 | -0.101 (-0.460, 0.258) | 0.580 | 0.0 |
| Study design | Cohort = 1; Cross-sectional = 0 | +0.659 (-0.193, 1.511) | 0.129 | 4.5 |
| Sex composition | Male-only = 1; Mixed/female = 0 | +0.053 (-0.257, 0.363) | 0.737 | 0.0 |

**Supplementary Table S10 –** Dose-response association of daily coffee intake (mL/day) and gout risk

| **Coffee Intake (mL/day)** | **OR** | **Lower95%CI** | **Upper95%CI** |
| --- | --- | --- | --- |
| **0.00** | 1.00 | 1.00 | 1.00 |
| **118.50** | 0.97 | 0.84 | 1.13 |
| **119.00** | 0.97 | 0.84 | 1.13 |
| **474.00** | 0.88 | 0.76 | 1.01 |
| **592.50** | 0.82 | 0.71 | 0.94 |
| **1066.50** | 0.56 | 0.44 | 0.71 |
| **1422.00** | 0.45 | 0.34 | 0.60 |
| **2133.00** | 0.38 | 0.19 | 0.74 |

**Supplementary Table S11 –** Dose-response association of daily coffee intake (mL/day) and hyperuricemia risk

| **Coffee Intake (mL/day)** | **OR** | **Lower95%CI** | **Upper95%CI** |
| --- | --- | --- | --- |
| **0.00** | 1.00 | 1.00 | 1.00 |
| **75.00** | 0.85 | 0.68 | 1.06 |
| **300.00** | 0.77 | 0.63 | 0.94 |
| **750.00** | 0.74 | 0.55 | 0.99 |
| **900.00** | 0.73 | 0.51 | 1.05 |
| **1575.00** | 0.77 | 0.43 | 1.39 |

**Supplementary Figure S30 –** Dose-response association of daily coffee intake (mL/day) and hyperuricemia risk in male

**Supplementary Table S12 –** Dose-response association of daily coffee intake (mL/day) and hyperuricemia risk in male

| **Coffee Intake (mL/day)** | **OR** | **Lower95%CI** | **Upper95%CI** |
| --- | --- | --- | --- |
| **0.00** | 1.00 | 1.00 | 1.00 |
| **75.00** | 0.91 | 0.83 | 0.99 |
| **300.00** | 0.77 | 0.63 | 0.94 |
| **750.00** | 0.75 | 0.57 | 1.00 |
| **1575.00** | 0.74 | 0.41 | 1.35 |

**Supplementary Figure S31 –** Dose-response association of daily coffee intake (mL/day) and hyperuricemia risk in female

**Supplementary Table S13 –** Dose-response association of daily coffee intake (mL/day) and hyperuricemia risk in female

| **Coffee Intake (mL/day)** | **OR** | **Lower95%CI** | **Upper95%CI** |
| --- | --- | --- | --- |
| **0.00** | 1.00 | 1.00 | 1.00 |
| **75.00** | 1.32 | 0.63 | 2.76 |
| **300.00** | 1.18 | 0.59 | 2.37 |
| **900.00** | 1.30 | 0.50 | 3.38 |

**Supplementary Table S14 –** Dose-response association of alcohol intake (g /day) and gout risk

| **Alcohol Intake**  **(g ethanol/day)** | **OR** | **Lower95%CI** | **Upper95%CI** |
| --- | --- | --- | --- |
| **0.00** | 1.00 | 1.00 | 1.00 |
| **2.50** | 1.00 | 0.99 | 1.02 |
| **7.45** | 1.14 | 1.10 | 1.18 |
| **12.45** | 1.50 | 1.43 | 1.56 |
| **22.45** | 1.94 | 1.86 | 2.01 |
| **39.95** | 2.39 | 2.23 | 2.56 |
| **75.00** | 3.21 | 2.84 | 3.63 |

**Supplementary Table S15 –** Dose-response association of daily alcohol intake (g /day) and hyperuricemia risk

| **Alcohol Intake**  **(g ethanol/day)** | **OR** | **Lower95%CI** | **Upper95%CI** |
| --- | --- | --- | --- |
| **0.00** | 1.00 | 1.00 | 1.00 |
| **7.50** | 1.04 | 0.93 | 1.17 |
| **15.00** | 1.10 | 0.97 | 1.25 |
| **22.50** | 1.17 | 1.04 | 1.32 |
| **30.00** | 1.26 | 1.11 | 1.42 |
| **45.00** | 1.46 | 1.27 | 1.67 |
| **75.00** | 2.03 | 1.34 | 3.06 |

**Supplementary Figure S32 –** Dose-response association of daily alcohol intake (g /day) and hyperuricemia risk in male

**Supplementary Table S16 –** Dose-response association of daily alcohol intake (g /day) and hyperuricemia risk in male

| **Alcohol Intake (g /day)** | **OR** | **Lower95%CI** | **Upper95%CI** |
| --- | --- | --- | --- |
| **0.00** | 1.00 | 1.00 | 1.00 |
| **12.50** | 1.08 | 0.95 | 1.22 |
| **14.95** | 1.10 | 0.96 | 1.25 |
| **15.00** | 1.10 | 0.96 | 1.25 |
| **30.00** | 1.26 | 1.11 | 1.42 |
| **37.00** | 1.35 | 1.19 | 1.53 |
| **45.00** | 1.46 | 1.28 | 1.67 |
| **75.00** | 2.00 | 1.32 | 3.03 |

**Supplementary Table S17 –** Dose-response association of daily sugar-sweetened beverage intake (mL/day) and gout risk

| **SSB Intake (mL/day)** | **OR** | **Lower95%CI** | **Upper95%CI** |
| --- | --- | --- | --- |
| **11.17** | 1.00 | 0.98 | 1.02 |
| **31.24** | 0.99 | 0.94 | 1.04 |
| **152.14** | 0.98 | 0.83 | 1.15 |
| **278.93** | 1.23 | 1.02 | 1.50 |
| **355.00** | 1.52 | 1.19 | 1.95 |
| **1065.00** | 1.95 | 1.35 | 2.82 |

**Supplementary Figure S33 –** Dose-response association of daily sugar-sweetened beverage intake (mL/day) and gout risk in male

**Supplementary Table S18 –** Dose-response association of daily sugar-sweetened beverage intake (mL/day) and gout risk in male

| **SSB Intake (mL/day)** | **OR** | **Lower95%CI** | **Upper95%CI** |
| --- | --- | --- | --- |
| **11.17** | 1.00 | 1.00 | 1.00 |
| **152.14** | 1.00 | 0.79 | 1.26 |
| **278.93** | 1.30 | 1.01 | 1.67 |
| **355.00** | 1.46 | 1.04 | 2.06 |
| **1065.00** | 1.86 | 1.10 | 3.15 |

**Supplementary Figure S34 –** Dose-response association of daily sugar-sweetened beverage intake (mL/day) and gout risk in female

**Supplementary Table S19 –** Dose-response association of daily sugar-sweetened beverage intake (mL/day) and gout risk in female

| **SSB Intake**  **(mL/day)** | **OR** | **Lower95%CI** | **Upper95%CI** |
| --- | --- | --- | --- |
| **11.17** | 1.00 | 1.00 | 1.00 |
| **31.24** | 1.04 | 0.95 | 1.15 |
| **152.14** | 0.96 | 0.80 | 1.15 |
| **278.93** | 1.16 | 0.91 | 1.48 |
| **355.00** | 1.69 | 1.19 | 2.39 |
| **1065.00** | 2.25 | 1.33 | 3.80 |

**Supplementary Table S20 –** Dose-response association of daily sugar-sweetened beverage intake (mL/day) and hyperuricemia risk

| **SSB Intake (mL/day)** | **OR** | **Lower95%CI** | **Upper95%CI** |
| --- | --- | --- | --- |
| **0.00** | 1.00 | 1.00 | 1.00 |
| **50.71** | 1.08 | 1.03 | 1.12 |
| **202.86** | 1.31 | 1.14 | 1.50 |
| **266.25** | 1.39 | 1.18 | 1.64 |
| **532.50** | 1.53 | 1.30 | 1.81 |
| **710.00** | 1.52 | 1.28 | 1.82 |
| **1597.50** | 1.72 | 1.41 | 2.08 |

**Supplementary Table S21 –** Dose-response association of tea intake (mL/day) and gout risk

| **Tea Intake(mL/day)** | **OR** | **Lower95%CI** | **Upper95%CI** |
| --- | --- | --- | --- |
| **0.00** | 1.00 | 1.00 | 1.00 |
| **119.00** | 1.07 | 0.94 | 1.22 |
| **592.50** | 0.98 | 0.84 | 1.15 |
| **1422.00** | 1.35 | 0.92 | 1.99 |

**Supplementary Table S22 –** Dose-response association of tea intake (occasions/week) and hyperuricemia risk

| **Tea Intake (occasions/week)** | **OR** | **Lower95%CI** | **Upper95%CI** |
| --- | --- | --- | --- |
| **0.00** | 1.00 | 1.00 | 1.00 |
| **1.13** | 0.99 | 0.94 | 1.03 |
| **3.13** | 0.98 | 0.92 | 1.05 |
| **3.50** | 0.98 | 0.92 | 1.05 |
| **4.50** | 0.98 | 0.93 | 1.05 |
| **9.00** | 0.99 | 0.94 | 1.05 |
| **10.50** | 1.00 | 0.94 | 1.06 |
| **31.50** | 1.00 | 0.92 | 1.09 |
